# Palmitate-Activated Macrophages Confer Insulin Resistance to Muscle Cells by a Mechanism Involving Protein Kinase C θ and ε

**DOI:** 10.1371/journal.pone.0026947

**Published:** 2011-10-26

**Authors:** Girish Kewalramani, Lisbeth Nielsen Fink, Farzad Asadi, Amira Klip

**Affiliations:** 1 Cell Biology Program, The Hospital for Sick Children, Toronto, Canada; 2 Hagedorn Research Institute, Novo Nordisk A/S, Gentofte, Denmark; 3 Department of Biochemistry, School of Veterinary Medicine, University of Tehran, Tehran, Iran; Hertie Institute for Clinical Brain Research and German Center for Neurodegenerative Diseases, Germany

## Abstract

**Background:**

Macrophage-derived factors contribute to whole-body insulin resistance, partly by impinging on metabolically active tissues. As proof of principle for this interaction, conditioned medium from macrophages treated with palmitate (CM-PA) reduces insulin action and glucose uptake in muscle cells. However, the mechanism whereby CM-PA confers this negative response onto muscle cells remains unknown.

**Methodology/Principal Findings:**

L6-GLUT4*myc* myoblasts were exposed for 24 h to palmitate-free conditioned medium from RAW 264.7 macrophages pre-treated with 0.5 mM palmitate for 6 h. This palmitate-free CM-PA, containing selective cytokines and chemokines, inhibited myoblast insulin-stimulated insulin receptor substrate 1 (IRS1) tyrosine phosphorylation, AS160 phosphorylation, GLUT4 translocation and glucose uptake. These effects were accompanied by a rise in c-Jun N-terminal kinase (JNK) activation, degradation of Inhibitor of κBα (IκBα), and elevated expression of proinflammatory cytokines in myoblasts. Notably, CM-PA caused IRS1 phosphorylation on Ser1101, and phosphorylation of novel PKCθ and ε. Co-incubation of myoblasts with CM-PA and the novel and conventional PKC inhibitor Gö6983 (but not with the conventional PKC inhibitor Gö6976) prevented PKCθ and ε activation, JNK phosphorylation, restored IκBα mass and reduced proinflammatory cytokine production. Gö6983 also restored insulin signalling and glucose uptake in myoblasts. Moreover, co-silencing both novel PKC θ and ε isoforms in myoblasts by RNA interference, but not their individual silencing, prevented the inflammatory response and restored insulin sensitivity to CM-PA-treated myoblasts.

**Conclusions/Clinical Significance:**

The results suggest that the block in muscle insulin action caused by CM-PA is mediated by novel PKCθ and PKCε. This study re-establishes the participation of macrophages as a relay in the action of fatty acids on muscle cells, and further identifies PKCθ and PKCε as key elements in the inflammatory and insulin resistance responses of muscle cells to macrophage products. Furthermore, it portrays these PKC isoforms as potential targets for the treatment of fatty acid-induced, inflammation-linked insulin resistance.

## Introduction

Low grade inflammation provoked by immune cells contributes to insulin resistance, a major cause of Type 2 diabetes [1], [2]. In obesity, macrophage infiltration of the expanding adipose tissue renders adipocytes insulin-resistant, consequently elevating circulating fatty acids and inflammatory cytokines that in turn contribute to insulin resistance in skeletal muscle [3], [4], [5], [6]. While the participation of macrophages in triggering adipose tissue insulin resistance is well established [3], [7], [8], it is less clear whether and how macrophage-derived factors affect skeletal muscle. Recent studies demonstrate the presence of proinflammatory macrophages infiltrating muscle tissue, whether abutting the skeletal muscle fibres directly or surrounding muscle-infiltrating adipocytes in the context of obesity [4], [5], [6], [9]. Cytokines released by local pro-inflammatory macrophages might directly affect muscle insulin action. Alternatively or concomitantly, cytokines produced by pro-inflammatory macrophages in the expanding adipose tissue may reach muscle in an endocrine fashion (via the circulation) to render it resistant to insulin. There is growing evidence that fatty acids trigger macrophages to secrete factors that impair insulin action [5], [10]. In this context, we and others have shown that fatty acids activate macrophages to release cytokines that cause insulin resistance in adipocytes and muscle cells [3], [5], [10], [11]. As proof of concept, we recently reported that conditioned medium from palmitate-treated macrophages (CM-PA) provokes insulin resistance in muscle cells at the levels of Akt signalling, glucose transporter 4 (GLUT4) translocation and glucose uptake [11], but how CM-PA confers this negative response onto muscle cells is yet unknown.

In addition to the cytokine-associated insulin resistance, the most abundant dietary saturated fatty acid palmitate can directly impair insulin signalling in muscle cells, reducing their insulin-dependent gain in surface GLUT4 and glucose uptake [12], [13], [14]. Several mechanisms have been proposed for the generation of palmitate-induced muscle insulin resistance, in particular the involvement of diacylglycerol (DAG)-sensitive novel protein kinase C's (PKCθ, PKCε, PKCδ, and PKCη) [15], [16], [17]. Palmitate and other fatty acids activate novel PKC's in skeletal muscle [16], [18], [19], thereby curbing downstream insulin signalling [15], [20], [21]. PKCθ can directly phosphorylate serine residues on Insulin Receptor Substrate-1 (IRS1) [20], and both PKCθ and PKCε also act upstream of the c-jun N-terminal kinase (JNK) to inhibit insulin signalling through this inflammatory stress kinase [22], [23], [24]. In addition, PKCε can phosphorylate the insulin receptor to reduce insulin action [25], [26]. Notably, PKCθ and PKCε also activate nuclear factor kappa B (NF-kB)-dependent pathways in muscle cells to promote expression of proinflammatory cytokines and chemokines, major mediators of inflammation-induced insulin resistance [16], [18]. Hence, novel PKC's intersect the muscle cell autonomous inflammatory response.

Given that both macrophage-derived factors and saturated fatty acids cause insulin resistance and activate muscle inflammatory pathways, and that fatty acids engage novel PKC's in this response, we hypothesized that this PKC family may represent a unifying sensor of dietary and inflammatory signals, that when activated would interfere with insulin action. Supporting this hypothesis, we report that CM-PA activates PKCθ and ε to induce insulin resistance at the levels of IRS1, Akt Substrate of 160 kDa (AS160), GLUT4 translocation and glucose uptake, along with the induction of pro-inflammatory pathways and increased expression of inflammatory cytokines within muscle cells. The results support the possibility that, *in vivo*, the ability of palmitate to induce insulin resistance may include activating macrophages to secrete proinflammatory cytokines and chemokines that would negatively impact on insulin action in skeletal muscle, acting through novel PKC's.

## Materials and Methods

### Materials and Reagents

Antibodies to phospho Akt (Ser473), phospho-AS160, phospho-JNK (Thr183/Tyr185), phospho-PKCθ (Thr538), phospho-IRS1 (Ser1101), phospho-NF-Kappa B (Ser536), NF-Kappa B (p65), GAPDH and IκBα were from Cell Signaling Technology (Danvers, MA). Monoclonal (9E10) and polyclonal (A-14) anti-*myc* antibodies, monoclonal anti-phosphotyrosine (PY99), antibodies to the insulin receptor, insulin receptor substrate (IRS)-1, phospho-IRS1 (Ser307), phospho-PKCε (Ser729), anti-histone and polyclonal anti-*myc* antibody were from Santa Cruz Biotechnology, Inc. (Santa Cruz, CA). PKC inhibitors Gö6983 and Gö6976 were from Calbiochem (San Diego, CA). Predesigned rat siPKCθ (5′-AACCTCAAGGCCGAATGCTAA-3′), siPKCε (5′-CACGATGAGTTCGTCACTGAT-3′) and non-related siRNA (siNR) (5′-AATAAGGCTATGAAGAGATAC-3′) were from Qiagen. Human insulin (Humulin R) was from Eli Lilly Canada (Toronto, Ontario, Canada). Cytochalasin B, 2-deoxyglucose (2-DG), protease inhibitor cocktail, monoclonal antiactinin-1, and other chemicals unless otherwise noted were from Sigma Chemical (St. Louis, MO). 2-Deoxy-D-[^3^H] glucose was from PerkinElmer (Boston, MA). The RNEasy Mini kit was from Qiagen (Mississauga, Ontario, Canada). The SuperScript® VILO™ cDNA Synthesis Kit was from Invitrogen (Burlington, Ontario, Canada). Reagents for quantitative PCR analysis were from Applied Biosystems (Carlsbad, CA). This includes Fast Advanced master mix and Taqman assays for detection of Hprt1 (Assay ID: Rn01527840_m1), tumor necrosis factor α (TNFα, Rn00562055_m1), interleukin 6 (IL6, Rn01410330_m1), Ptgs2 (COX2, Rn01483828_m1) and Ccl2 (MCP1, Rn00580555_m1) transcripts. The Proteome Profiler Mouse Cytokine Array Kit, Panel A and recombinant mouse cytokines/chemokines (CXCL1, CXCL2, CXCL10 and TNFα) were from RnD Systems (Minneapolis, MN).

### L6-GLUT4myc myoblast cell lines and treatments

L6-GLUT4*myc* myoblasts [27] without or with stable expression of the Akt substrate AS160 (L6-GLUT4*myc*-AS160 cells) or of the insulin receptor (L6-GLUT4*myc*-IR cells), as indicated, were cultured in α-MEM supplemented with 10% FBS, blasticidin S (2 µg/ml), and 1% antibiotic/antimycotic solution (10,000 U/ml penicillin G, 10 mg/ml streptomycin, and 25 µg/ml amphotericin B) under 5% CO_2_ at 37°C. Myoblast cultures were typically treated with the indicated macrophage conditioned media (CM) or exogenous cytokines for the times indicated, serum-deprived for 3 h and stimulated with insulin (100 nM, 20 min). Where listed, Gö6983 (1 µM), or Gö6976 (1 µM) were used to inhibit PKC's.

### Macrophage treatment with palmitate and collection of conditioned media (CM)

RAW 264.7 macrophages (purchased from American Type Culture Collection, Manassas, VA, USA, Catalogue No TIB-71) were grown in α-MEM supplemented with 1% antibiotic-antimycotic and 10% fetal bovine serum. A 5 mM palmitate-10% BSA stock solution was prepared as described [12]. Macrophages were treated with BSA alone or 500 µM conjugated palmitate/1% BSA in culture medium for 6 h, then washed several times with PBS, and fresh medium was added. After 12 h, conditioned media (CM-BSA, CM-PA) were collected, centrifuged (7 min, 3,500 rpm), and added to L6-GLUT4*myc* myoblast cultures for 1 h or 24 h. Given the intermediate washes, no residual palmitate is expected to be present in CM-PA. Following the 1 h or 24 h incubation, cells were washed twice with 0.5 ml PBS and lysed in the appropriate ice-cold lysis buffer. Cell lysates were used for immunoblotting, immunoprecipitation and gene expression analysis.

### RT-PCR and small interfering RNA (siRNA)-mediated PKC knockdown

For quantitative PCR (qPCR), RNA was isolated with the RNEasy Mini kit and used to generate cDNA using The SuperScript® VILO™ cDNA Synthesis Kit (Invitrogen). qPCR was performed using Fast Advanced master mix (Applied Biosystems) and Taqman assays for rat TNFα, IL6, monocyte chemotactic protein 1 (MCP-1) and cyclooxygenase 2 (COX-2) according to the manufacturer's instructions. The input of cDNA was 10 ng/reaction. qRT-PCR was performed at 50°C/2 min, 95°C/20 sec followed by 40 cycles of 95°C/1 sec and 60°C/20 sec. Comparative cycle threshold results expressed relative to hprt1 were used. For PKCθ and PKCε knockdown, L6GLUT4*myc* myoblasts were treated with 100 nM siRNA to PKCθ (PKCθ siRNA) (AACCTCAAGGCCGAATGCTAA) or siRNA to PKCε (PKCε siRNA) (CACGATGAGTTCGTCACTGAT) or a combination of both at 100 nM each using lipofectamine RNAi-max reagent (Invitrogen).

### Immunoblotting and immunoprecipitation

Confluent L6-GLUT4*myc* myoblasts in 10 cm diameter dishes were treated as indicated and immediately processed for SDS-PAGE and immunoblotting as described [28]. IRS1 in cell lysates was immunoprecipitated using an anti-IRS1 antibody. The immunocomplex and the supernatant were resuspended in Laemmli buffer and heated for 5 min at 95°C. The immunocomplex was separated into two equal portions, separated by SDS-PAGE and immunoblotted with anti-phosphotyrosine, antiphospho-IRS1 (Ser307) or antiphospho-IRS1 (Ser1101), and anti-IRS1.

### Nuclear localization of Nuclear Factor-Kappa B (NF-kappa B)

A nuclear extract kit (Active Motif, CA) was used to obtain cytosolic and nuclear fractions from L6 myoblasts. Briefly, after treatments, cells were scraped and centrifuged for 5 min at 500 rpm at 4°C. resuspended in hypotonic 1× buffer and detergent-extracted. The suspension was centrifuged for 30 s at 14,000 g at 4°C, yielding the cytoplasmic fraction in the supernatant and a nuclear pellet that was re-purified. Samples were analyzed for phosphorylated or total NF-Kappa B/p65 (1∶1000).

### 2-deoxy-[3H]glucose uptake and cell surface GLUT4*myc*

L6-GLUT4*myc* myoblasts grown in 24-well plates and serum-deprived for 3–5 h, then treated with or without insulin (100 nM, 20 min). 2-Deoxyglucose uptake was measured as described [28], and cell surface GLUT4*myc* was measured as described [27], [29].

### Cytokine and chemokine determination and treatment

CM-PA & CM-BSA were analyzed for multiple cytokines and chemokines by Proteome Profiler Mouse Cytokine Array (RnD Systems) according to the manufacturer's instructions for cell culture supernatants. Membranes were exposed to autoradiography film for 1 s, 30 s or 2 min. Protein spots were quantified using ImageJ software (NIH, USA). To examine the impact of cytokines and chemokines on activation of PKCθ and ε, myoblasts were incubated with cytokines and chemokines (0–100 ng/ml) individually or in combination, for 24 h. Subsequently cells were washed and lysed in the appropriate ice-cold lysis buffer prior to immunoblotting.

### Measurement of non-esterified fatty acids (NEFAs) in media

NEFA levels were measured according to the ACS.ACOD (Acyl CoA synthase - Acyl CoA oxidase) method using the HR series NEFA-HR kit No. 999-34691 (Wako Pure Chemical Industries, Osaka, Japan).

### Statistical analysis

Values are means ± SE. Wherever appropriate, one-way ANOVA followed by Tukey or Bonferroni tests was used to determine differences between group mean values. The level of statistical significance was set at *P*<0.05.

## Results

### CM-PA increases IRS1 serine phosphorylation and reduces its tyrosine phosphorylation in response to insulin

**Figure 1:**
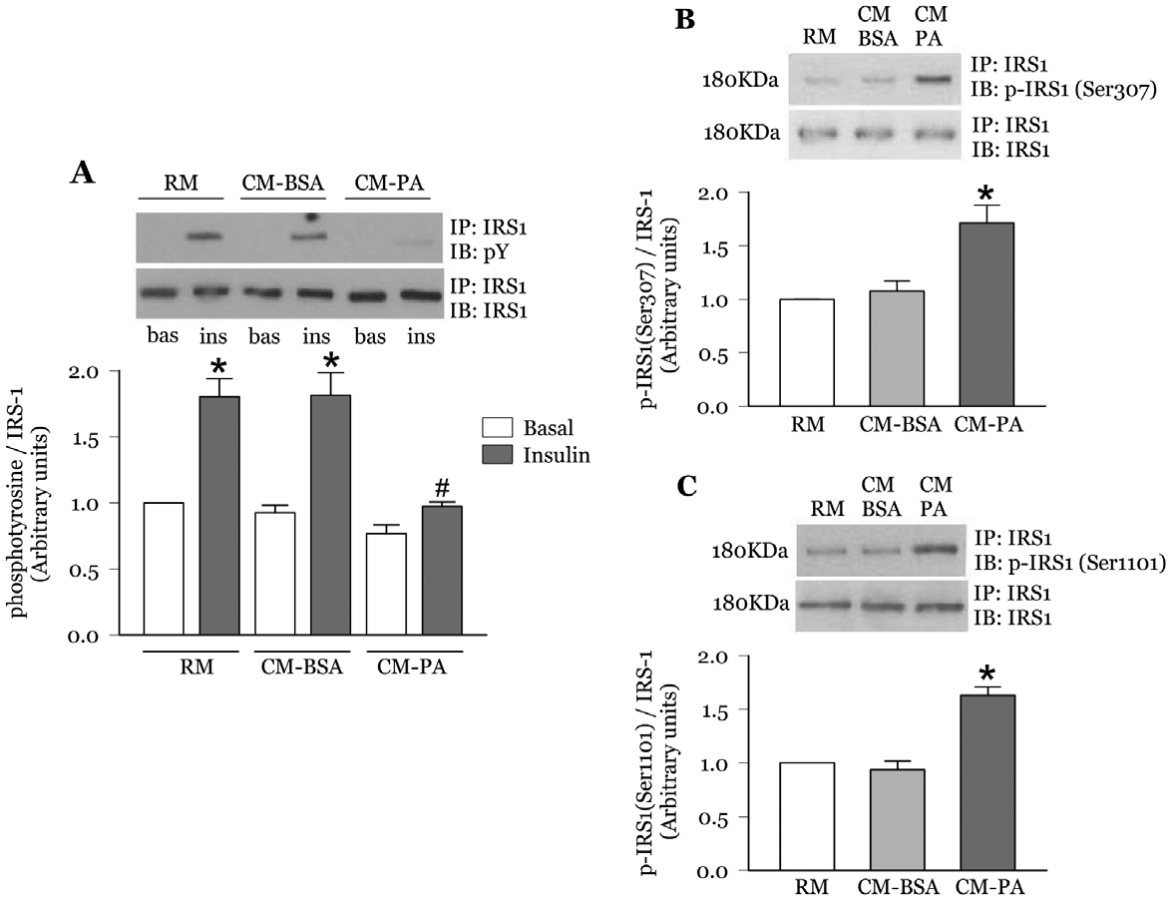
CM-PA impairs insulin stimulated tyrosine phosphorylation of IRS-1 in L6-GLUT4*myc* myoblasts. L6-GLUT4*myc* myoblasts were incubated for 24 h with RM, CM-BSA or CM-PA from RAW macrophages. Cells were lysed, immunoprecipitated using IRS-1 antibody and subjected to SDS-PAGE either directly or following stimulation with insulin (100 nM for 10 min). **A;** Representative immunoblots and densitometric quantification for tyrosine phosphorylated IRS-1 (pY) relative to total IRS-1.Bas, Basal; Ins, Insulin treated; p, phosphorylated. Results are mean ± SE of 3 separate experiments. *Significantly different from basal, ^#^Significantly different from CM-BSA insulin, *P*<0.05. **B;** Representative immunoblots and densitometric quantification for IRS-1 phosphorylation at Ser307 and **C;** Ser1101 relative to total IRS-1. Results are mean ± SE of 3 separate experiments. *Significantly different from CM-BSA, *P*<0.05.

We have previously reported a drop in insulin-stimulated phosphorylation of Akt on Ser473 in L6 myoblasts treated with ceramides as a model of lipotoxicity [30] or with conditioned medium from palmitate-treated macrophages (CM-PA) as a model of inflammatory toxicity [11]. The ceramide effect was not accompanied by upstream signalling defects at the level of IRS1, the substrate of the insulin receptor relevant for GLUT4 traffic [31]. However, a defect at this first step in insulin action is often observed in conditions of *in vivo* insulin resistance. Hence we first assessed whether macrophage-conditioned media alters insulin signalling at the level of IRS1. In myoblasts incubated with medium from untreated macrophages (regular medium, RM) or CM-BSA, insulin increased tyrosine phosphorylation of IRS1 by approximately 2 fold (Fig. 1A). On the other hand, in myoblasts treated with CM-PA, insulin-stimulated tyrosine phosphorylation of IRS1 was significantly reduced (45±6%, Fig. 1A). No change in IRS1 tyrosine phosphorylation was observed in the absence of insulin. Phosphorylation of IRS1 on selective serine residues can reduce its tyrosine phosphorylation in response to insulin [32]. Indeed, when compared with CM-BSA, CM-PA significantly increased myoblast IRS1 phosphorylation at serine 307 and 1101 (1.7±0.2-fold *vs* CM-BSA and 1.6±0.1-fold vs CM-BSA, Fig. 1B and 1C).

### CM-PA activates novel PKCθ and PKCε in L6 myoblasts

**Figure 2:**
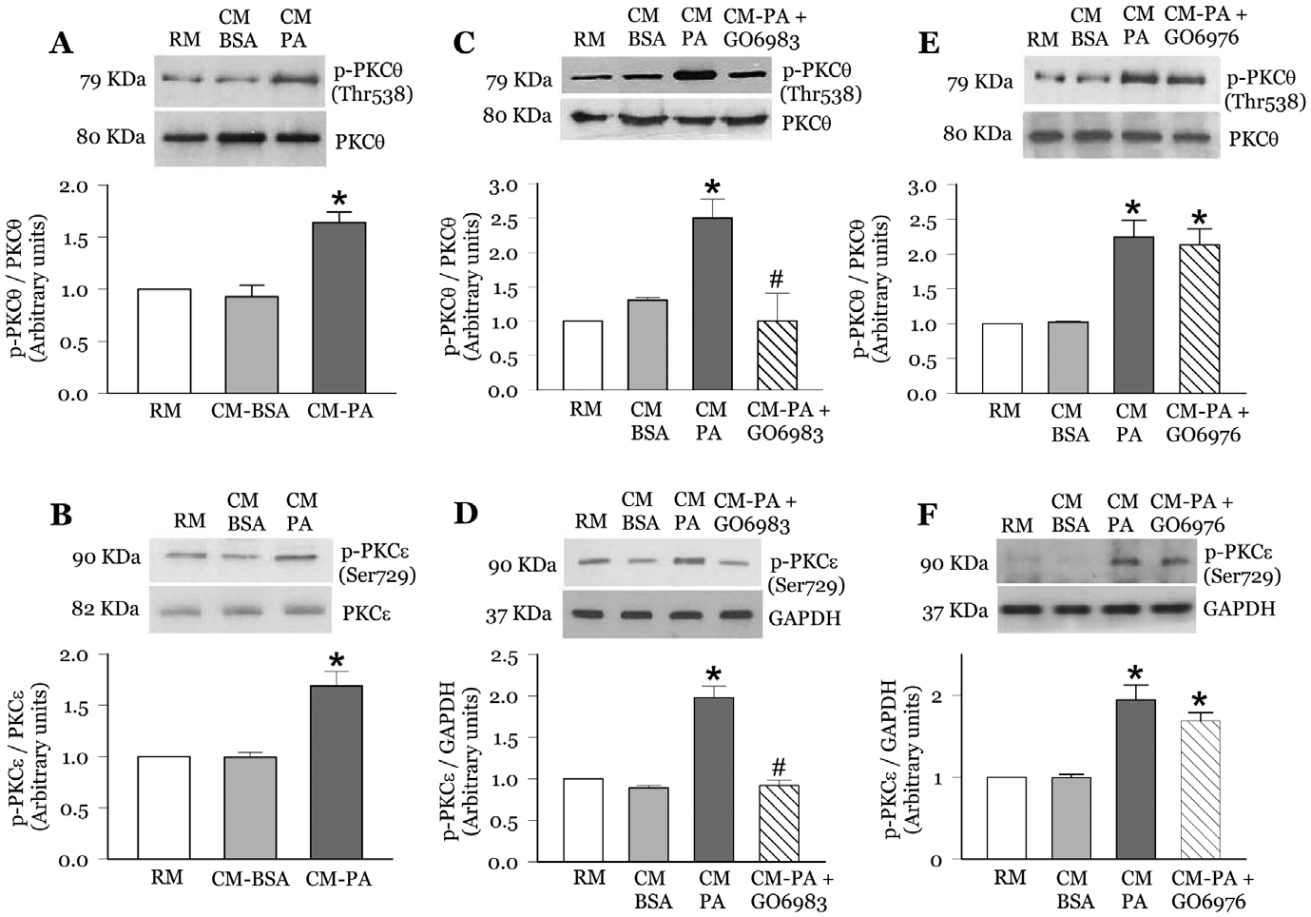
CM-PA causes activation of novel PKCθ and PKCε in L6-GLUT4*myc* myoblasts. L6-GLUT4*myc* myoblasts were treated for 24 h with RM, CM-BSA and CM-PA. Where indicated, cells were pre-incubated for 30 min with the novel and conventional PKC inhibitor, Gö6983 (1 µM) or the conventional PKC inhibitor, Gö6976 (1 µM), prior to addition of CM-PA. After 24 h, cells were lysed, and equal amount of total protein from each sample were immunoblotted with specific antibodies against phospho-Thr538 PKCθ, total PKCθ (**A,** **C** and **E**), phospho-Ser729 PKCε, total PKCε or GAPDH (**B**, **D** and **F**). Representative immunoblots and densitometric quantification of three experiments are shown. Values are expressed as ratio of phosphorylated protein relative to GAPDH or total levels of respective protein. Results are mean ± SE. *Significantly different from CM-BSA, *P*<0.05. ^#^Significantly different from CM-PA, *P*<0.05.

Pro-inflammatory cytokines and chemokines activate PKCθ and ε [33], [34], and these kinases can lead to IRS1 serine phosphorylation thereby reducing its tyrosine phosphorylation and downstream activation of Akt [15], [20], [21]. Since we have observed elevated levels of the cytokine TNFα in CM-PA [11], we next explored whether CM-PA activates novel PKC's in myoblasts. PKCθ is activated by phosphorylation of Thr538 in its activation loop, and PKCε through phosphorylation of Ser729 within its carboxyl-terminal hydrophobic site [35], [36], [37], [38]. We found that while incubating myoblasts for 24 h with RM or CM-BSA had no effect on either PKC phosphorylation, CM-PA caused an approximate 1.6-fold rise in each PKCθ Thr538 and PKCε Ser729 phosphorylation, without changing either PKC protein content (Fig. 2A and 2B). Of note, incubating myoblasts with CM-BSA or CM-PA for an acute period of 1 h had no effect on either PKC phosphorylation (Fig. S1). To verify if the CM-PA-induced PKCθ and PKCε activation influenced insulin signalling, we first used the novel and conventional PKC inhibitor Gö6983 and the conventional PKC inhibitor Gö6976. Gö6983 or Gö6976 had no effect on myoblast PKCθ or PKCε phosphorylation in unchallenged cells (data not shown). In the presence of CM-PA, Gö6983 abrogated the increase in PKCθ and PKCε phosphorylation (Fig. 2C and Fig. 2D). As expected, Gö6976 did not alter the CM-PA-induced PKCθ or PKCε activation (Fig. 2E and 2F).

### Inhibition of novel PKC's prevents the reduction in insulin-dependent IRS1 tyrosine phosphorylation provoked by CM-PA

**Figure 3:**
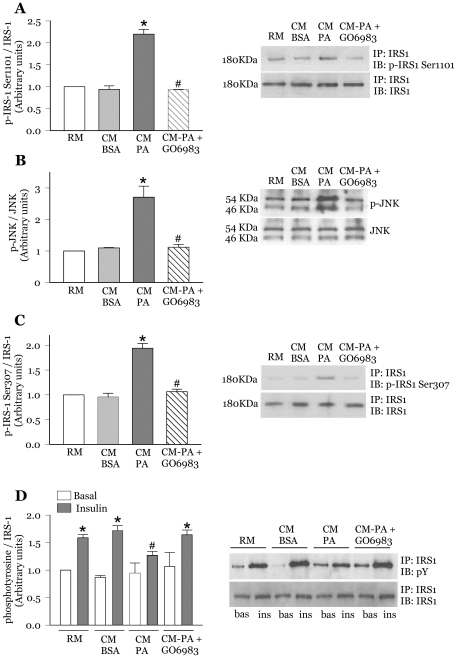
Novel PKC inhibition prevents the JNK activation and drop in insulin-stimulated tyrosine phosphorylation of IRS1 caused by CM-PA. L6-GLUT4*myc* myoblasts were treated for 24 h with RM, CM-BSA and CM-PA. Where indicated, cells were pre-incubated for 30 mins with the novel and conventional PKC inhibitor, Gö6983 (1 µM), prior to addition of CM-PA. Cells were lysed, immunoprecipitated using IRS-1 antibody and subjected to SDS-PAGE either directly or following stimulation with insulin (100 nM for 10 min). Representative immunoblots and quantification of phosphorylation of Ser1101-IRS1 (**A**), Ser307-IRS1 (**C**) and tyrosine residues (**D**). For direct immunoblotting, cells were lysed, and equal amount of total protein from each sample were immunoblotted with specific antibodies against phospho-JNK and total JNK (**B**). Representative immunoblots and quantification of three to four experiments are shown. Values are expressed as ratio of phosphorylated protein relative to total levels of respective protein. Results are mean ± SE *Significantly different from basal or CM-BSA, *P*<0.05, ^#^Significantly different from CM-BSA insulin or CM-PA, *P*<0.05.

Phosphorylation of IRS1 at a number of susceptible serine residues lowers insulin signalling by reducing its insulin-stimulated tyrosine phosphorylation. In particular, PKCθ can directly induce phosphorylation of IRS1 at serine 1101 in muscle [20]. There is no known, direct PKCε-dependent IRS1 phosphorylation although both PKCθ and PKCε can affect IRS1 acting through JNK [22], [23], [24], and PKCε can modulate insulin receptor function and attenuate downstream insulin signalling [25], [26]. Given that CM-PA activated both PKC's, we investigated whether the deleterious actions of CM-PA on IRS1 were averted by the novel and conventional PKC inhibitor Gö6983. Indeed, Gö6983 abrogated the CM-PA-induced increase in phosphorylation of IRS1 at Ser1101 without change in the total content of IRS1 (Fig. 3A). In contrast, although CM-PA reduced the insulin dependent tyrosine phosphorylation of the insulin receptor, this response was not restored by Gö6983 (Fig. S2). We previously documented an increase in c-jun N-terminal kinase (JNK) phosphorylation in myoblasts treated with CM-PA [11]. As both PKCθ and PKCε can also act via JNK to increase IRS1 phosphorylation at Ser307 [22], [23], [24], we assessed whether novel PKC inhibition prevented CM-PA-induced JNK and IRS1 Ser307 phosphorylation. Indeed, myoblast treatment with Gö6983 markedly reduced both these effects of CM-PA, without changing the total JNK or IRS1 content (Fig. 3B and 3C). Importantly, the Gö6983-induced loss of IRS1 serine phosphorylation was accompanied by restoration of insulin induced IRS1 tyrosine phosphorylation (Fig. 3D). Of note, the conventional PKC inhibitor Gö6976 failed to restore the CM-PA-mediated drop in IRS1 tyrosine phosphorylation (data not shown). These results suggest that the effects on IRS1 caused by CM-PA are mediated through its activation of PKCθ and PKCε.

### Gö6983 reverses the drop in insulin-stimulated Akt and AS160 phosphorylation evoked by CM-PA

**Figure 4:**
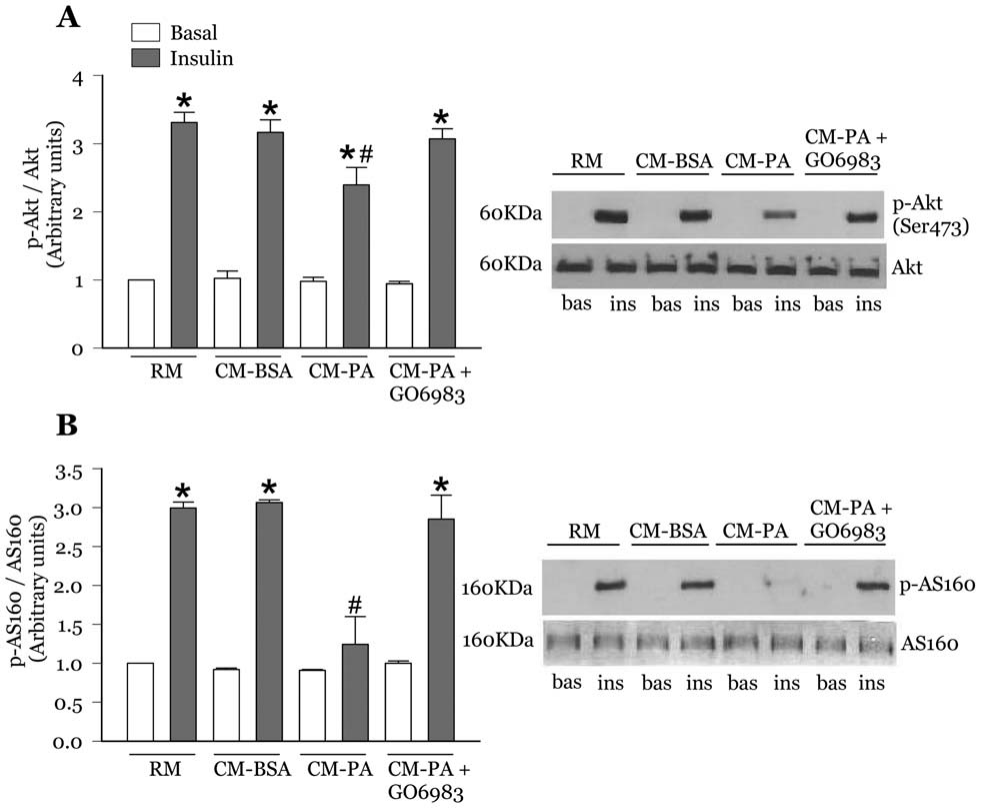
Novel PKC inhibition prevents the decrease in insulin-stimulated phosphorylation of Akt and AS160 caused by CM-PA. L6-GLUT4*myc* myoblasts with or without stable expression of AS160 (L6-GLUT4*myc*-AS160) were incubated as described in Fig. 3, and then stimulated in the presence or absence of insulin (100 nM, 10 min). Cells were lysed, and equal amount of total protein from each sample were immunoblotted with specific antibodies against phospho-Akt (Ser473) and total Akt (**A**), or phospho-AS160 and total AS160 (**B**). Representative immunoblots and quantification of three experiments are shown. Values are expressed as ratio of phosphorylated protein relative to total levels of the respective protein. Results are the means ± SE *Significantly different from basal, *P*<0.05, ^#^Significantly different from CM-BSA insulin, *P*<0.05.

Downstream of IRS1, phosphoinositide 3-kinase (PI3K) activates Akt that in turn phosphorylates the RAB-GAP AS160, to allow activation of Rabs 8A and 13 that are essential for GLUT4 translocation in muscle cells [39]. Exposing myoblasts to CM-PA for 24 h reduced insulin-stimulated phosphorylation of Akt on Ser473 (Fig. 4A) and importantly caused a more robust drop in AS160 phosphorylation, signifying severe impairment in downstream insulin signalling (Fig. 4B). Consistent with the lack of stimulation of PKCs, comparable levels of insulin-stimulated Akt and AS160 phosphorylation were observed in myoblasts incubated with CM-PA for an acute period of 1 h (Fig. S3), suggesting that a long-term incubation is required for CM-PA to induce impairment in insulin signalling. Given that Gö6983 prevented the long-term, deleterious effects of CM-PA on IRS1, we explored whether downstream signalling was similarly restored. Indeed, this novel and conventional PKC inhibitor abolished the negative impact of CM-PA and restored insulin-stimulated Akt (Fig. 4A) and AS160 phosphorylation (Fig. 4B) without affecting basal state phosphorylation. In contrast, the conventional PKC inhibitor Gö6976 did not prevent either effect of CM-PA (data not shown).

### Novel PKC inhibition prevents the reduction of GLUT4*myc* translocation and glucose uptake caused by CM-PA

**Figure 5:**
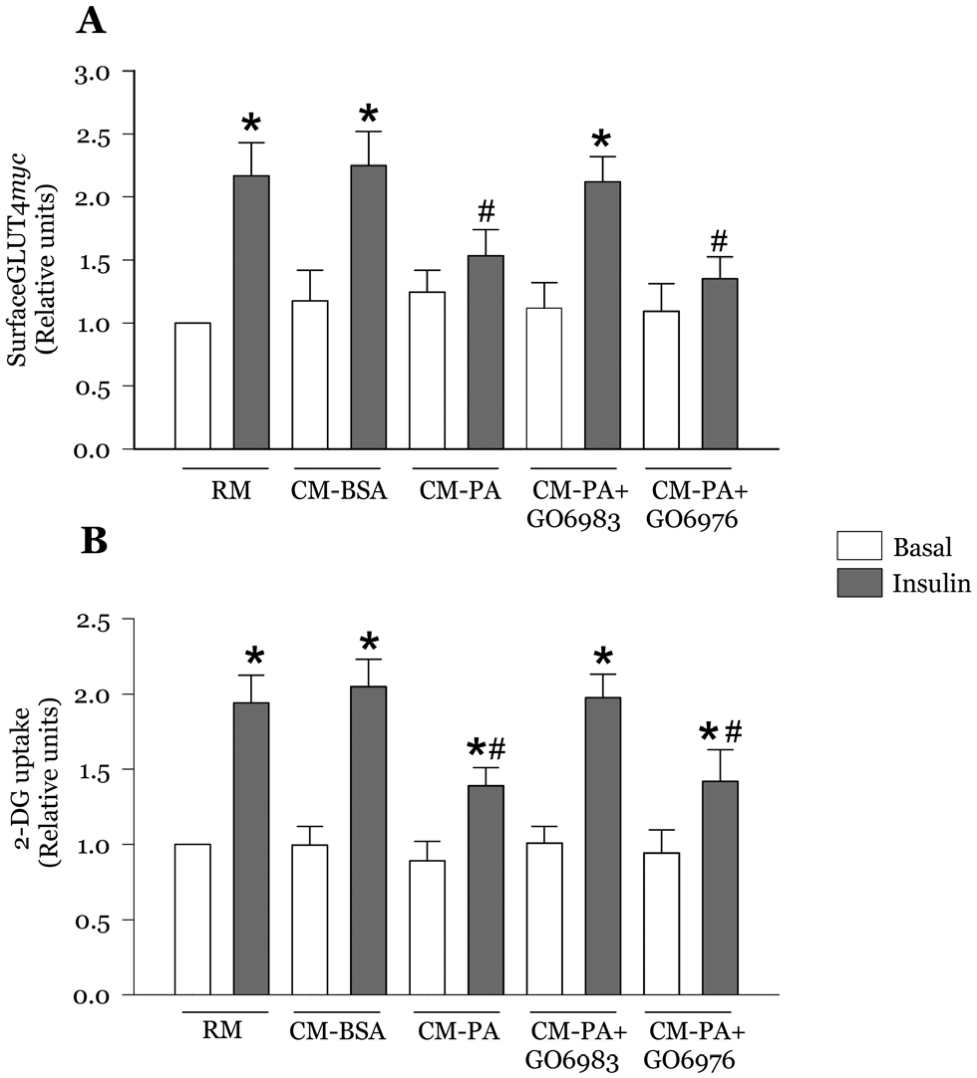
Novel PKC inhibition blunts the deleterious effect of CM-PA on insulin-stimulated GLUT4 exocytosis and glucose uptake. L6-GLUT4*myc* myoblasts were treated for 24 h with RM, CM-BSA, CM-PA, or CM-PA supplemented with Gö6983 (1 µM). Following 2 h of serum starvation, myoblasts were stimulated in the presence or absence of insulin (100 nM for 20 min) and then assayed for cell surface density of GLUT4*myc* (**A**) or 2-DG uptake (**B**). Data are presented as the means ± SE of 3 experiments. *Significantly different from basal, *P*<0.05, ^#^Significantly different from CM-BSA insulin, *P*<0.05.

Insulin action through IRS1, Akt and AS160 stimulates glucose uptake by increasing GLUT4 glucose transporters at the plasma membrane of muscle cells [39]. In myoblasts incubated with CM-PA, insulin-stimulated gain in surface GLUT4*myc* translocation (Fig. 5A) and glucose uptake (Fig. 5B) were significantly reduced when compared with CM-BSA, confirming our previous observations [11]. Notably, the novel PKC inhibitor Gö6983 prevented the CM-PA- induced insulin resistance of GLUT4*myc* translocation and its consequent glucose uptake, such that the decreases in either parameter below the insulin-stimulated CM-BSA were no longer statistically significant (Fig. 5A and B). In contrast, Gö6976 did not restore insulin action on these metabolic outcomes (Fig. 5A and B).

### Specific cytokines and chemokines in macrophage-conditioned media fail to activate novel PKC's in muscle cells

**Figure 6:**
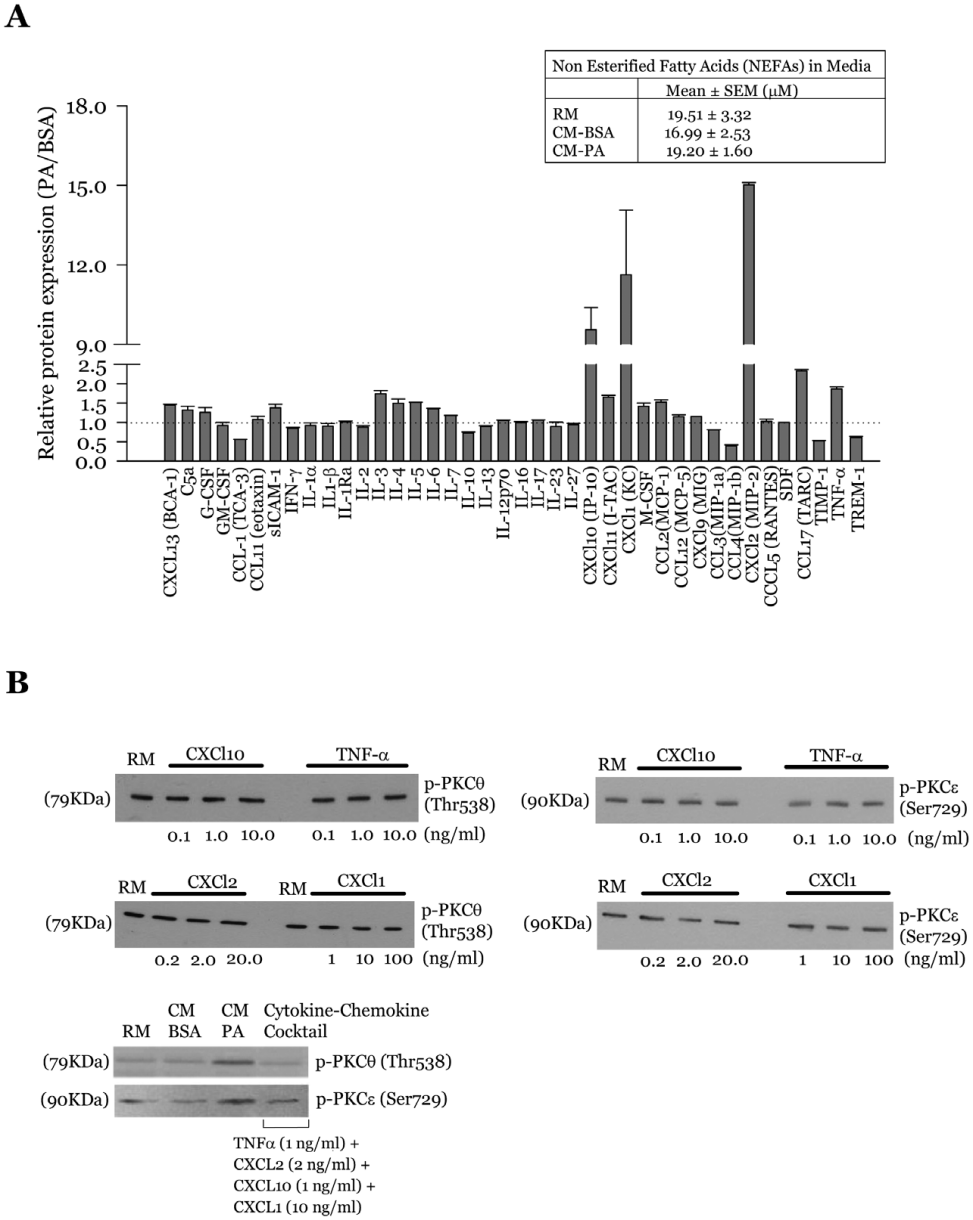
Specific cytokines/chemokines identified in conditioned media fail to activate novel PKC's in muscle cells. **A,** Palmitate treatment induces differential secretion of cytokines and chemokines by macrophages, revealed through a Proteome Profiler Mouse Cytokine Array. Values are expressed as the ratio of expression in CM-PA over CM-BSA, and represent the means ± SE of 2 separate experiments. NEFA levels were measured using HR series NEFA-HR kit No. 999-34691 (inset). Values are presented as the means ± SE of 3 experiments. **B,** L6-GLUT4*myc* myoblasts were incubated individually or in combination with different concentrations (0–100 ng/ml) of TNFα, MIP2, IP10 or KC for 24 h. Cells were lysed, and equal amount of protein from each sample were immunoblotted with specific antibodies against phospho-PKCθ (Thr538) and phospho-PKCε (Ser729). Representative immunoblots of three experiments are shown.

Several studies have shown that macrophage-derived cytokines and chemokines such as TNFα and MCP-1 can directly elicit adipose cell inflammation and insulin resistance [40], [41]. Accordingly, it is likely that secreted cytokines in the conditioned media from palmitate-treated macrophages propagate inflammatory stress response and insulin resistance in muscle cells. Our previous studies showed elevated levels of TNFα in CM-PA compared to CM-BSA [11]. A more detailed analysis of this conditioned media in the present study validated that observation and further demonstrated markedly elevated levels of MCP-1 (CCL2), MIP2 (CXCL2), IP10 (CXCL10), KC (CXCL1) and CCL7 (Fig. 6A). Besides macrophage-derived factors, residual palmitate or fatty acids potentially released by the macrophages might also be present in CM-PA, even though palmitate was removed after 6 h, macrophages washed several times with PBS and fresh medium added for 12 h to collect conditioned media. To investigate these possibilities, we measured the levels of non-esterified free fatty acids (NEFA) in CM-PA. Fig. 6A (inset) shows that there was no difference in NEFA's in CM-PA compared to CM-BSA, and moreover the levels detected fall below those reported in the literature to affect insulin action in muscle [12]. This result suggests that a protein component is likely involved in the observed defect in myoblast insulin signalling. To examine the individual impact of small protein molecules like cytokines and chemokines on activation of PKC theta and epsilon, myoblasts were incubated individually for 24 h with different concentrations (0–100 ng/ml) of the most differentially elevated cytokines and chemokines found in CM-PA compared to CM-BSA: TNFα, MIP2, IP10 and KC. Interestingly, no effect on PKCθ or PKCε phosphorylation was observed in muscle cells challenged individually with these cytokines or chemokines at indicated concentrations (Fig. 6B). We further tested the effect these cytokines and chemokines combined, using the following concentrations: TNFα (1 ng/ml), MIP2 (2 ng/ml), IP10 (1 ng/ml) and KC (10 ng/ml). However, like the individual cyto/chemokine treatments, the combination treatment also failed to elicit any rise in PKCθ and PKCε phosphorylation (Fig. 6B).

### Macrophage-conditioned media causes IΚBα degradation, promotes NF-kappa B nuclear translocation and elevates the expression of pro-inflammatory cytokines

**Figure 7:**
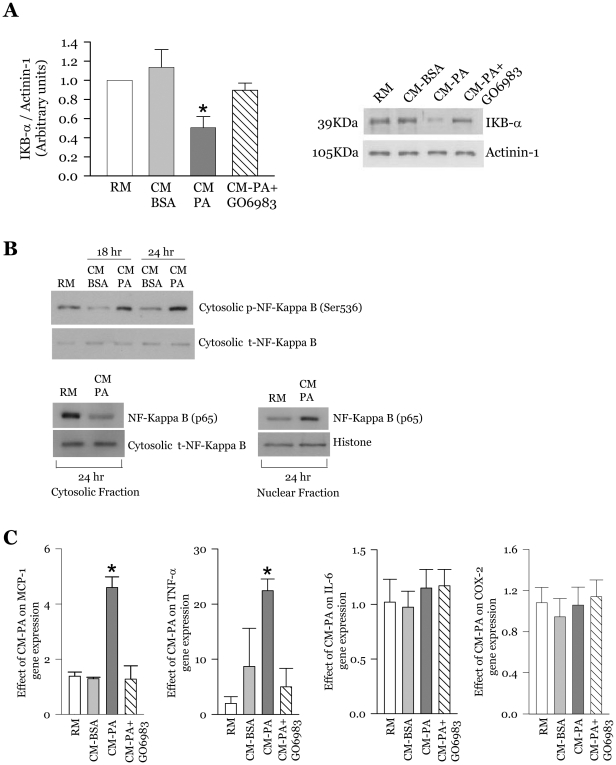
CM-PA promotes IκBα degradation, NF-kappa B nuclear translocation and induces the expression of cytokines and chemokines in myoblasts. **A,** Representative immunoblots and densitometric quantification of IκBα and actinin-1 after exposure of L6-GLUT4myc myoblasts to RM, CM-BSA, CM-PA, or CM-PA supplemented with Gö6983 (1 µM). Actinin-1 served as internal loading control, and values are expressed as IκBα to actinin-1 ratio. Results are the mean ± SE of three independent experiments. **B,** L6-GLUT4*myc* myoblasts were treated for 18 or 24 h with RM, CM-BSA and CM-PA, and cytoplasmic and nuclear fractions were obtained. Cells were lysed, and equal amount of protein from each sample were immunoblotted with specific antibodies against phosphorylated NF-kappa B (pSer536) and total NF-kappa B/p65. Histone and total NF-kappa B were used as controls. Representative immunoblots and quantification of three experiments are shown. **C,** Representative densitometric quantification of transcript levels of MCP-1, TNFα, IL-6 and COX-2. Results are the mean ± SE of three independent experiments. *Significantly different from all other groups, *P*<0.05.

Muscle can mount an autonomous inflammatory stress response to cytokines and chemokines, evinced by degradation of IΚBα and the consequent activation of NF-kB dependent gene transcription through post-translational modifications of the NF-kB p65 subunit [42], [43], [44], [45]. Consistent with this behaviour, we previously reported that CM-PA promotes IΚBα degradation and elevates the expression of proinflammatory cytokines in the target myoblasts [11]. As PKCθ and PKCε have been demonstrated to act upstream of IΚBα [46], [47], we here hypothesized that inhibition of novel PKC's would prevent the CM-PA-driven IΚBα degradation and subsequent responses. This was found to be the case, as Gö6983 prevented IΚBα degradation by 81±9% (Fig. 7A) in myoblasts incubated with CM-PA for 24 h, and further significantly reduced the CM-PA dependent augmentation in MCP-1 and TNFα gene expression (Fig. 7C). Interestingly, there was no significant change in myoblast IL-6 or COX-2 gene expression in myoblasts treated with CM-PA (Fig. 7C), indicating that the effects of CM-PA are selective rather than the result of a generalized inflammatory response by the muscle cells. To directly confirm the activation of NF-kB, the phosphorylation status of its main p65 phosphorylation site, Ser536, was examined. Incubation of myoblasts with CM-PA for up to 24 h increased the cytosolic phosphorylation of NF-κB p65 at Ser536 starting at 18 h and peaking at 24 h (Fig. 7B). Further, nuclear translocation is crucial for NF-κB to exert its transcriptional effects. We found that 24 h of CM-PA treatment stimulated NF-κB nuclear translocation (Fig. 7B).

### Co-silencing PKCθ and PKCε expression prevents the inhibition of insulin signalling caused by CM-PA

**Figure 8:**
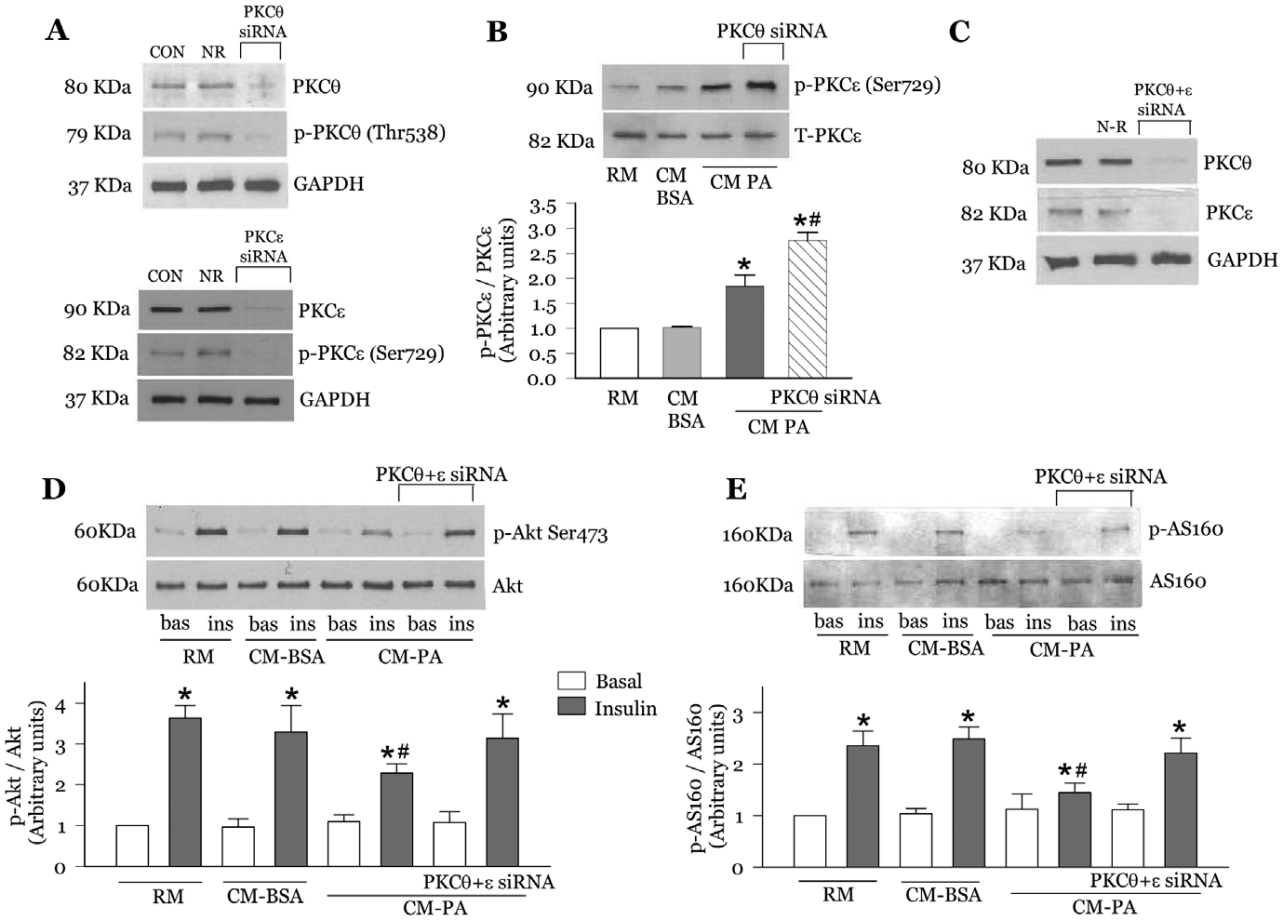
Novel PKC silencing reverses CM-PA mediated drop in insulin-stimulated phosphorylation of Akt and AS160. L6-GLUT4myc myoblasts were incubated with non-related siRNA (NR), siRNA to PKCθ (PKCθ siRNA), siRNA to PKCε (PKCε siRNA) or a combination of both (PKCθ+ε) using lipofectamine RNAi-max reagent. **A** and **C**, knockdown efficiency PKCθ and PKCε, respectively. **B**, L6-GLUT4myc myoblasts were treated for 24 h with RM, CM-BSA, CM-PA or CM-PA supplemented with siRNA to PKCθ (PKCθ siRNA). After 24 h, cells were lysed and each sample was immunoblotted with specific antibodies against phospho-PKCε (Ser729) and total-PKCε. Results are the means ± SE of three separate experiments *Significantly different from CM-BSA, *P*<0.05, ^#^Significantly different from CM-PA, *P*<0.05. **D** and **E**, L6-GLUT4myc myoblasts with or without stable expression of AS160 (L6-GLUT4*myc*-AS160) were treated for 24 h with RM, CM-BSA, CM-PA or CM-PA supplemented with siRNA to both PKCθ and PKCε. Following 24 h treatment, cells were stimulated in the presence or absence of insulin (100 nM, 10 min). Cells were lysed, and equal amount of total protein from each sample were immunoblotted with specific antibodies against phospho-Akt (Ser473), total Akt, phospho-AS160 and total AS-160. Results are mean ± SE of three separate experiments *Significantly different from basal, *P*<0.05, ^#^Significantly different from CM-BSA insulin, *P*<0.05.

The results described so far revealed that palmitate-treated macrophages produce a selective array of cytokines and chemokines into a complex CM-PA, which in turn unleashes insulin resistance and inflammatory responses within muscle cells. These responses were largely ascribed to novel PKC activation, based on the alleviation of these deleterious effects by Gö6983 but not Gö6976. To specifically ascertain that CM-PA indeed acted through PKCθ and PKCε in its ability to alter insulin action, we aimed to silence PKCθ and PKCε expression in myoblasts via siRNA. We first validated successful PKCθ and PKCε knockdown by immunoblotting (Fig. 8A). Knockdown of either PKCθ or PKCε alone by their corresponding siRNA did not prevent the reduction in insulin-stimulated phosphorylation of Akt or AS160 induced by CM-PA (Fig. S4 A & B). Interestingly, however, knockdown of PKCθ alone enhanced by 31±4% the activating phosphorylation of PKCε observed with CM-PA (Fig. 8B). As this suggested a redundant or perhaps cooperative action of these proteins in their ability to interfere with insulin action, we enquired whether silencing both proteins together could overcome the deleterious effects of CM-PA on muscle cells. Myoblasts were transfected with a mix of siRNAs to both PKCθ and PKCε prior to challenging them with CM-PA. Proving our hypothesis, reducing PKCθ protein content by 74±4% (Fig. 8C) and PKCε content by 76±3% (Fig. 8C) in this manner virtually abolished the inhibition in insulin-stimulated phosphorylation of Akt (Fig. 8D) and AS160 (Fig. 8E) provoked by CM-PA.

### Co-silencing PKCθ and PKCε expression prevents the effect of CM-PA on myoblast GLUT4*myc* translocation and glucose uptake

**Figure 9:**
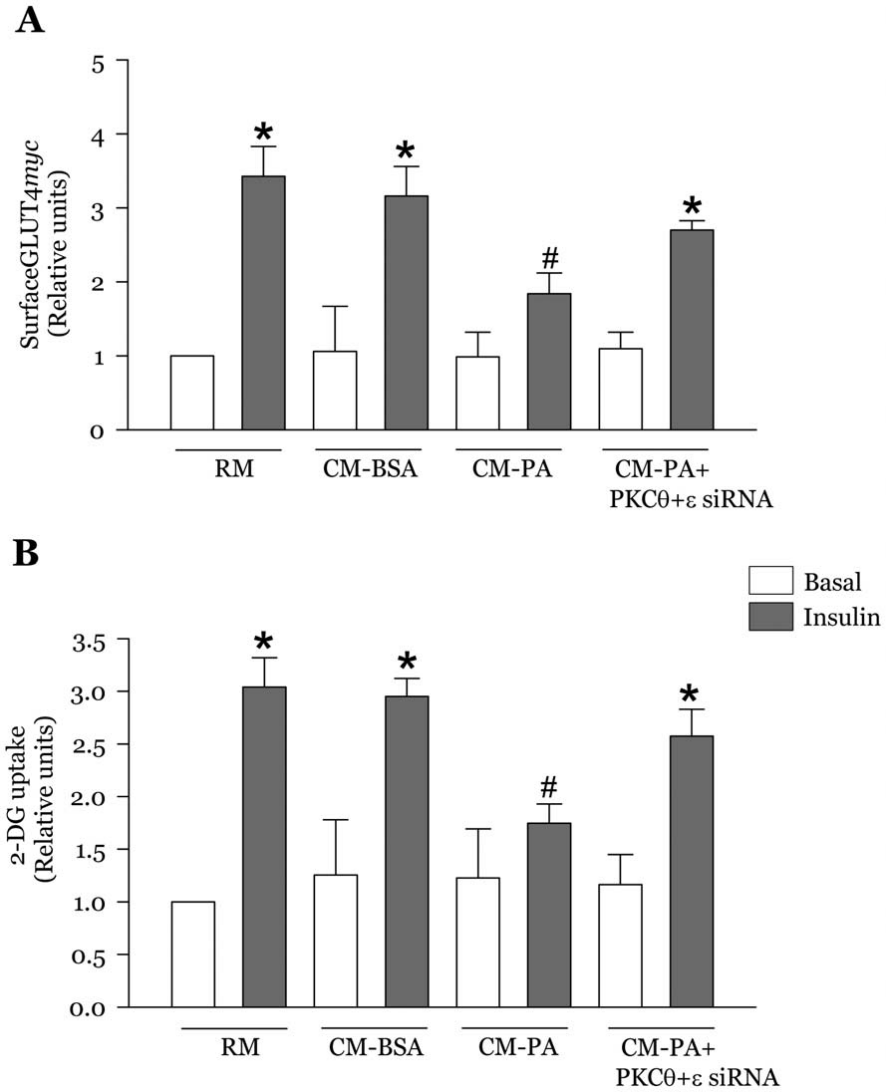
Co-silencing novel PKCθ and PKCε prevented the deleterious effect of CM-PA on insulin-stimulated GLUT4 translocation and glucose uptake. L6-GLUT4*myc* myoblasts were treated for 24 h with RM, CM-BSA, CM-PA, or CM-PA supplemented with siRNA to both PKCθ and PKCε (100 nM each). Following 2 h of serum starvation, myoblasts were stimulated in the presence or absence of insulin (100 nM for 20 min) and then assayed for cell surface density of GLUT4*myc* (**A**) or 2-deoxyglucose uptake (**B**). Data are presented as means ± SE of 3 experiments. *Significantly different from basal, *P*<0.05, ^#^Significantly different from CM-BSA insulin, *P*<0.05.

We finally sought to ascertain that the metabolic consequence of the macrophage-derived media on muscle cells was specifically mediated by PKCθ and PKCε. Indeed, co-silencing both PKC isoforms relieved insulin resistance, by preventing the drop in insulin stimulated GLUT4*myc* translocation (Fig. 9A) and glucose uptake (Fig. 9B) caused by CM-PA.

## Discussion

Resident macrophages are an essential component of skeletal muscle. Positive functions of macrophages include contribution to regeneration and revascularization when in their M2 (anti-inflammatory) phenotypic polarization [1]. However, macrophages can also become M1 polarized (inflammatory), which results in inflammatory cytokine production [1]. In cell culture, products from inflammatory macrophages can confer insulin resistance to muscle cells, as in the case of fatty acid-treated RAW macrophages and L6 myoblasts [1], [5], [11]. However, the signalling mechanism by which macrophage-derived factors confer this negative response onto muscle cells is poorly understood. The present study shows that conditioned media from palmitate-treated macrophages (CM-PA) activates novel PKCθ and PKCε that appear to be responsible for induction of pro-inflammatory gene expression, cytokine production and metabolic insulin resistance in muscle cells. Since only very low levels of NEFA's were detected in the conditioned media and these levels did not differ between CM-PA and CM-BSA, it is unlikely that any residual fatty acid is responsible for the activation of these kinases in the muscle cells.

An interplay between fatty acids and macrophages to promote muscle cell insulin resistance is now recognized. Previous studies have shown that conditioned medium from fatty acid-activated bone marrow-derived macrophages reduces glucose uptake in muscle cells [5]. Building on those observations, we have shown that the saturated fatty acid palmitate polarizes macrophages of the RAW264.7 cell line towards the inflammatory M1 phenotype. Moreover, CM-PA from such macrophages renders L6 muscle cells insulin-resistant [11]. In the present study, an analysis of CM-PA revealed markedly elevated levels of selective cytokines and chemokines such as TNFα, MCP-1, MIP2, IP10, and KC. Notably, TNFα, MCP-1, IP10, and KC have been shown to activate PKC's and induce inflammatory stress via NF-κB [33], [45], [48], [49], [50]. Interestingly, we found no effect on PKCθ or PKCε phosphorylation in muscle cells challenged with these cytokines or chemokines, individually or in combination, suggesting that other macrophage-released products or their combinations are responsible for the biological effects of CM-PA. Future analysis will make it possible to dissect out whether and which other chemokines/cytokines are responsible for the insulin resistance and the inflammatory responses conferred to muscle cells.

### Novel PKC's reduce insulin signalling at the level of IRS1

PKC activation is one of several pathways leading to Ser/Thr phosphorylation of IRS proteins, mainly IRS1 [51]. Serine phosphorylation of IRS1 at a number of susceptible sites attenuates downstream insulin signalling by reducing its insulin-stimulated tyrosine phosphorylation. Studies suggest that phosphorylation of mouse IRS1 at Ser307 (Ser312 in human IRS1) is important for inflammatory stimuli-mediated inhibition of IRS1 function [20]. Here we document an increase in IRS1 Ser307 phosphorylation in muscle cells exposed to CM-PA. Activation of novel PKC's may not mediate Ser307 phosphorylation directly, since this site is not recognized as a direct target of these kinases, yet inhibiting novel PKC's prevented this phosphorylation. This finding may be explained by the action of both PKCθ and PKCε upstream of JNK [22], [23], [24], the kinase directly responsible for Ser307 phosphorylation [52]. In addition, CM-PA caused IRS1 phosphorylation at Ser1101, a site that can be directly phosphorylated by novel PKCθ in muscle cells and tissue [20]. Because Ser1101 phosphorylation has been consequently linked to reduced IRS-1 tyrosine phosphorylation and downstream insulin signalling [20], we hypothesized that PKCθ may be directly responsible for the reduction in IRS1 signalling in muscle cells challenged with CM-PA. Indeed, we report that chemical inhibition of novel PKC's or silencing gene expression of PKCθ and PKCε prevents both IRS1 Ser1101 phosphorylation and tyrosine phosphorylation.

Besides IRS1, CM-PA treatment of myoblasts reduced the insulin-dependent tyrosine phosphorylation of the insulin receptor. More than one mechanism may be responsible for this effect. Pro-inflammatory cytokine administration to muscle cells increases the activity of protein-tyrosine phosphatase 1B (PTP1B) that dephosphorylates phosphotyrosine residues in the insulin receptor [53]. It is therefore possible that inflammatory factors present in CM-PA contribute to the observed reduction in insulin receptor tyrosine phosphorylation via phosphatase like PTP1B. Serine phosphorylation of IRS1, via a feedback loop, might also inhibit insulin receptor tyrosine kinase activity [54], which may explain the decrease in insulin receptor tyrosine phosphorylation observed with CM-PA (however, this would not be mediated by IRS1 phosphorylation on Ser307 or Ser1101, see below).

### CM-PA acts through novel PKC's to reduce Akt and AS160 responses to insulin

Down-stream of IRS1, activation of Akt by insulin requires hierarchical phosphorylation on two residues, Thr308 and Ser473 [55]. CM-PA treatment of myoblasts resulted in a small reduction in insulin-stimulated phosphorylation of Akt at Ser473. It is possible that this mild reduction in Akt may not suffice *per se* to impose GLUT4 resistance downstream, since studies show that much greater reductions are required for this metabolic outcome [55], [56], [57]. However, the more marked inhibition of AS160 phosphorylation observed with CM-PA reveals an amplification of insulin resistance and may be the direct cause of the impaired insulin-stimulated GLUT4 translocation and glucose uptake. Like novel PKC's, activation of conventional PKC isoforms (α, βI, βII, and γ) is associated with inhibition of early steps in the insulin signalling cascade [58], [59]. As Gö6983 (novel and conventional PKC inhibitor) but not Gö6976 (specific conventional PKC inhibitor), restored insulin signalling through the IRS1, Akt and AS160 leading to normalization of muscle glucose uptake, our findings suggest that the CM-PA-induced block in insulin action downstream of the insulin receptor is mediated by novel PKC's, likely PKCθ and ε. Failure of Gö6983 to prevent the drop in insulin receptor tyrosine phosphorylation advocates that the effect of CM-PA on the insulin receptor is mediated by a mechanism not involving novel PKC's. Further, since Gö6983 restored the impairment of downstream insulin signalling in attendance of reduced insulin receptor tyrosine phosphorylation, it can be concluded that the partial alteration in insulin receptor function has no consequence on downstream insulin action.

### CM-PA elicits inflammatory responses in muscle cells mediated by novel PKC's

Activation of the NF-κB signalling cascade is pivotal to inflammatory responses and insulin resistance in muscle cells [60]. In the resting state, IκBα proteins restrict the NF-κB dimer in the cytoplasm. Phosphorylation of IκBα by IκB kinase induces its proteasomal degradation, releasing the NF-κB heterodimer to translocate to the nucleus and regulate proinflammatory gene expression [60]. Interestingly, we found that Gö6983 prevented the IκBα degradation and induction of inflammatory cytokine/chemokine (TNFα and MCP1) gene expression in muscle cells caused by CM-PA. Hence we propose that the inflammatory response of muscle cells challenged by CM-PA is mediated through novel PKC's. These results add to a growing number of studies showing involvement of novel PKC's in cell autonomous inflammation: In pancreatic acinar cells, TNFα activates novel PKCδ and ε to regulate NF-κB mediated gene transcription [33]. In the human eosinophilic leukemia cell line EoL-1, MCP-1 activates PKCδ to promote an NF-κB mediated inflammatory response [45]. Direct treatment of C2C12 muscle cells with palmitate elevates IL-6 gene expression via activation of NF-κB and PKC and concomitantly reduces insulin signalling [61]. Palmitate also activates NF-κB and promotes pro-inflammatory cytokine expression by a mechanism involving novel PKC's in C2C12 and L6 skeletal muscle cells [16], [18]. Hence, fatty acids and cytokines alike, such as those present in CM-PA, activate novel PKC's in muscle cells to in turn cause insulin resistance.

### Identification of PKCθ and PKCε as the mediators of CM-PA-induced inflammation and insulin resistance in muscle cells

A major goal of this study was to identify the PKC isoforms responsible for CM-PA- induced insulin resistance. Because isoform specific pharmacological inhibitors are not currently available for most PKC's, the most convincing way to demonstrate a causative role for a particular isoform in the generation of insulin resistance is to compare effects in cells transfected with isoform-specific siRNA sequences. Co-silencing both PKCθ and ε by RNA interference (but not individual PKC silencing) prevented the inflammatory response and restored insulin sensitivity to CM-PA-treated muscle cells. These results suggest that among novel PKC's, the PKCθ and ε isoforms may be critical for CM-PA-induced inflammation and insulin resistance. Further, single knockdown of PKCθ is compensated by increased activation of PKCε in CM-PA treated myoblasts, suggesting that an additional role for PKCε in muscle cells is unmasked.

### PKCθ and PKCε involvement in insulin resistance *in vivo*

PKCθ and PKCε have emerged as strong candidates for mediating lipid-induced insulin resistance. In mice, PKCθ inactivation prevents lipid infusion-induced defects in insulin signalling and glucose transport in skeletal muscle [62]. In addition, knockout of each PKCθ or PKCε genes in mice enhances insulin signalling and glucose tolerance, and protect against fat induced insulin resistance [62], [63]. This collective evidence points to a pivotal role for these novel PKC's in insulin resistance *in vivo*. Our results add a new dimension to this connection, by suggesting the possibility that, in the context of high fat diets or fatty acid infusion, cytokines are released by pro-inflammatory macrophages (activated by adipose tissue-derived or circulating fatty acids), which in turn impinge on muscle, to activate PKCθ and PKCε in muscle cells. We speculate that activation of these novel PKC isoforms may be a consequence of diacylglycerol formation in response to pro-inflammatory factors present in CM-PA, since there is precedent that cytokine treatment promotes diacylglycerol formation in muscle cells [34]. Finally, our findings reveal that silencing either PKC isoform alone would not be a suitable pharmacological strategy to overcome inflammation and insulin resistance in the context of high fat diets, and instead suggest that combined inhibition of both PKCθ and ε would be far more beneficial.

**Figure 10:**
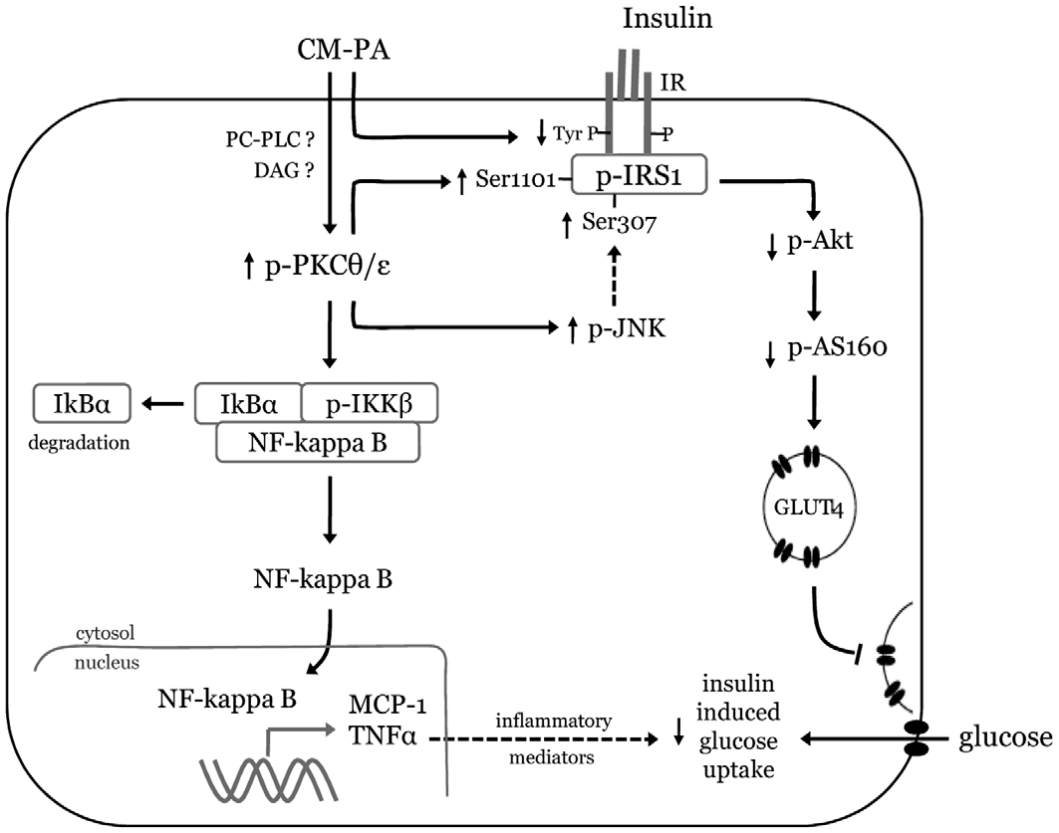
Proposed mechanisms involved in the effects of macrophage-derived factors on muscle cells. Cytokines in macrophage-conditioned medium (CM-PA) impinge on muscle cells to activate novel PKCθ and PKCε. Activation of these novel PKC isoforms may be a consequence of diacylglycerol (DAG) formation in response to pro-inflammatory factors present in CM-PA. PKCθ and ε activation stimulated Ser1101 phosphorylation on IRS-1 directly and Ser307 on IRS-1 via PKC-mediated JNK activation. Serine phosphorylated of IRS-1 reduced phosphotyrosine and this is associated with reduced insulin signaling to Akt and AS160 and GLUT4 exocytosis to the plasma membrane and glucose uptake. PKC activation also mediates NF-kappa B activation (via IκBα degradation) and pro-inflammatory gene expression (e.g. TNFα and MCP-1). The release of these factors may act in autocrine and paracrine modes to further impair insulin-stimulated glucose uptake.

In summary, we report that that the block in muscle insulin action brought about by CM-PA is mediated by the novel PKCθ and PKCε. Both isoforms, directly and/or via the stress kinase JNK inhibit insulin signalling at the level of IRS1, Akt and AS160 lowering insulin-dependent GLUT4 translocation and glucose uptake (Fig. 10). CM-PA treatment of skeletal muscle cells also induces pro-inflammatory gene expression through the activation of PKCθ/ε-NF-κB axis, which likely contributes further to the decline in insulin action (Fig. 10). In the future, we aim to identify which of the various inputs of PKC's dominates in mediating the CM-PA-induced insulin resistance in muscle cells. Overall, this study re-establishes the participation of macrophages as a relay in the action of fatty acids on muscle cells, and further identifies PKCθ and PKCε as key elements in the inflammatory and insulin resistance responses of muscle cells to macrophage products. Furthermore, it portrays these PKC isoforms as a potential joint target for the treatment of fatty acid-induced, inflammation-linked insulin resistance.

## Supporting Information

Figure S1 **Acute incubation of muscle cells with CM-PA fails to activate novel PKC's.** Macrophages were treated with BSA alone or 500 µM palmitate/1% BSA in culture medium for 6 h, then washed several times with PBS, and fresh medium was added. After 12 h, conditioned media (CM-BSA, CM-PA) were collected, centrifuged, and added to myoblast cultures for 1 h. As a control, myoblasts were simultaneously incubated with exogenous TNF-alpha (10 ng/ml for 1 h or 24 h). Lysates were prepared from all conditions and equal amounts of protein from each sample were immunoblotted with specific antibodies against phospho-PKCθ (Thr538) and phospho-PKCε (Ser729). Representative gels of two experiments are shown.
(TIF)

Figure S2 **Novel PKC inhibition does not prevent the drop in insulin-stimulated tyrosine phosphorylation of the insulin receptor observed with CM-PA.** L6-GLUT4*myc* cells expressing the human insulin receptor were treated for 24 h with RM, CM-BSA and CM-PA. Where indicated, cells were pre-incubated for 30 min with the novel and conventional PKC inhibitor, Gö6983 (1 µM), prior to addition of CM-PA. Cells were lysed and equal amount of total protein from each sample were immunoblotted with anti-pY or anti-IR β-subunit either directly or following stimulation with insulin (100 nM for 10 min). Representative gels of two experiments are shown.
(TIF)

Figure S3 **Short-term incubation of muscle cells with CM-PA does not impact on insulin-stimulated phosphorylation of Akt and AS160.** Macrophages were treated with BSA alone or 500 µM palmitate/1% BSA in culture medium for 6 h, and then washed several times with PBS, and fresh medium was added. After 12 h, conditioned media (CM-BSA, CM-PA) were collected, centrifuged, and added to myoblast cultures for 1 h, then myobalsts were serum-starved and pretreated with or without insulin (100 nM, 20 min) as usual. As a control, myoblasts were simultaneously incubated with exogenous TNF-alpha (10 ng/ml for 1 h). Lysates were prepared and equal amount of protein from each sample were immunoblotted with specific antibodies against phospho-Akt (Ser473) and phospho-AS160. Representative gels of two experiments are shown.
(TIF)

Figure S4 **Individually silencing novel PKCθ or ε does not restore insulin sensitivity in CM-PA treated myoblasts.** L6-GLUT4*myc* myoblasts were treated for 24 h with RM, CM-BSA, CM-PA, or CM-PA supplemented with siRNA to PKCθ or PKCε (100 nM each). Following 2 h of serum starvation, myoblasts were stimulated in the presence or absence of insulin (100 nM for 20 min). Cells were lysed, and equal amount of total protein from each sample were immunoblotted with specific antibodies against phospho-Akt (Ser473) and total Akt (**A**), or phospho-AS160 and total AS-160 (**B**). Representative immunoblots of two experiments are shown.
(TIF)

## References

[1] Olefsky JM, Glass CK (2010). Macrophages, inflammation, and insulin resistance.. Annu Rev Physiol.

[2] Heilbronn LK, Campbell LV (2008). Adipose tissue macrophages, low grade inflammation and insulin resistance in human obesity.. Curr Pharm Des.

[3] Lumeng CN, Deyoung SM, Saltiel AR (2007). Macrophages block insulin action in adipocytes by altering expression of signaling and glucose transport proteins.. Am J Physiol Endocrinol Metab.

[4] Weisberg SP, McCann D, Desai M, Rosenbaum M, Leibel RL (2003). Obesity is associated with macrophage accumulation in adipose tissue.. J Clin Invest.

[5] Nguyen MT, Favelyukis S, Nguyen AK, Reichart D, Scott PA (2007). A subpopulation of macrophages infiltrates hypertrophic adipose tissue and is activated by free fatty acids via Toll-like receptors 2 and 4 and JNK-dependent pathways.. J Biol Chem.

[6] Varma V, Yao-Borengasser A, Rasouli N, Nolen GT, Phanavanh B (2009). Muscle inflammatory response and insulin resistance: synergistic interaction between macrophages and fatty acids leads to impaired insulin action.. Am J Physiol Endocrinol Metab.

[7] Lumeng CN, Deyoung SM, Bodzin JL, Saltiel AR (2007). Increased inflammatory properties of adipose tissue macrophages recruited during diet-induced obesity.. Diabetes.

[8] Lumeng CN, Bodzin JL, Saltiel AR (2007). Obesity induces a phenotypic switch in adipose tissue macrophage polarization.. J Clin Invest.

[9] Vettor R, Milan G, Franzin C, Sanna M, De Coppi P (2009). The Origin of Intermuscular Adipose Tissue and Its Pathophysiological Implications.. Am J Physiol Endocrinol Metab.

[10] Hevener AL, Olefsky JM, Reichart D, Nguyen MT, Bandyopadyhay G (2007). Macrophage PPAR gamma is required for normal skeletal muscle and hepatic insulin sensitivity and full antidiabetic effects of thiazolidinediones.. J Clin Invest.

[11] Samokhvalov V, Bilan PJ, Schertzer JD, Antonescu CN, Klip A (2009). Palmitate- and lipopolysaccharide-activated macrophages evoke contrasting insulin responses in muscle cells.. Am J Physiol Endocrinol Metab.

[12] Dimopoulos N, Watson M, Sakamoto K, Hundal HS (2006). Differential effects of palmitate and palmitoleate on insulin action and glucose utilization in rat L6 skeletal muscle cells.. Biochem J.

[13] Holland WL, Brozinick JT, Wang LP, Hawkins ED, Sargent KM (2007). Inhibition of ceramide synthesis ameliorates glucocorticoid-, saturated-fat-, and obesity-induced insulin resistance.. Cell Metab.

[14] Alkhateeb H, Chabowski A, Glatz JF, Luiken JF, Bonen A (2007). Two phases of palmitate-induced insulin resistance in skeletal muscle: impaired GLUT4 translocation is followed by a reduced GLUT4 intrinsic activity.. Am J Physiol Endocrinol Metab.

[15] Yu C, Chen Y, Cline GW, Zhang D, Zong H (2002). Mechanism by which fatty acids inhibit insulin activation of insulin receptor substrate-1 (IRS-1)-associated phosphatidylinositol 3-kinase activity in muscle.. J Biol Chem.

[16] Barma P, Bhattacharya S, Bhattacharya A, Kundu R, Dasgupta S (2009). Lipid induced overexpression of NF-kappaB in skeletal muscle cells is linked to insulin resistance.. Biochim Biophys Acta.

[17] Dey D, Mukherjee M, Basu D, Datta M, Roy SS (2005). Inhibition of insulin receptor gene expression and insulin signaling by fatty acid: interplay of PKC isoforms therein.. Cell Physiol Biochem.

[18] Jove M, Planavila A, Sanchez RM, Merlos M, Laguna JC (2006). Palmitate induces tumor necrosis factor-alpha expression in C2C12 skeletal muscle cells by a mechanism involving protein kinase C and nuclear factor-kappaB activation.. Endocrinology.

[19] Dey D, Basu D, Roy SS, Bandyopadhyay A, Bhattacharya S (2006). Involvement of novel PKC isoforms in FFA induced defects in insulin signaling.. Mol Cell Endocrinol.

[20] Li Y, Soos TJ, Li X, Wu J, Degennaro M (2004). Protein kinase C Theta inhibits insulin signaling by phosphorylating IRS1 at Ser(1101).. J Biol Chem.

[21] Mack E, Ziv E, Reuveni H, Kalman R, Niv MY (2008). Prevention of insulin resistance and beta-cell loss by abrogating PKCepsilon-induced serine phosphorylation of muscle IRS-1 in Psammomys obesus.. Diabetes Metab Res Rev.

[22] Ghaffari-Tabrizi N, Bauer B, Villunger A, Baier-Bitterlich G, Altman A (1999). Protein kinase Ctheta, a selective upstream regulator of JNK/SAPK and IL-2 promoter activation in Jurkat T cells.. Eur J Immunol.

[23] Werlen G, Jacinto E, Xia Y, Karin M (1998). Calcineurin preferentially synergizes with PKC-theta to activate JNK and IL-2 promoter in T lymphocytes.. Embo J.

[24] Brandlin I, Eiseler T, Salowsky R, Johannes FJ (2002). Protein kinase C(mu) regulation of the JNK pathway is triggered via phosphoinositide-dependent kinase 1 and protein kinase C(epsilon).. J Biol Chem.

[25] Ikeda Y, Olsen GS, Ziv E, Hansen LL, Busch AK (2001). Cellular mechanism of nutritionally induced insulin resistance in Psammomys obesus: overexpression of protein kinase Cepsilon in skeletal muscle precedes the onset of hyperinsulinemia and hyperglycemia.. Diabetes.

[26] Kellerer M, Mushack J, Mischak H, Haring HU (1997). Protein kinase C (PKC) epsilon enhances the inhibitory effect of TNF alpha on insulin signaling in HEK293 cells.. FEBS Lett.

[27] Ueyama A, Yaworsky KL, Wang Q, Ebina Y, Klip A (1999). GLUT-4myc ectopic expression in L6 myoblasts generates a GLUT-4-specific pool conferring insulin sensitivity.. Am J Physiol.

[28] Niu W, Huang C, Nawaz Z, Levy M, Somwar R (2003). Maturation of the regulation of GLUT4 activity by p38 MAPK during L6 cell myogenesis.. J Biol Chem.

[29] Wang Q, Khayat Z, Kishi K, Ebina Y, Klip A (1998). GLUT4 translocation by insulin in intact muscle cells: detection by a fast and quantitative assay.. FEBS Lett.

[30] JeBailey L, Wanono O, Niu W, Roessler J, Rudich A (2007). Ceramide- and oxidant-induced insulin resistance involve loss of insulin-dependent Rac-activation and actin remodeling in muscle cells.. Diabetes.

[31] Huang C, Thirone AC, Huang X, Klip A (2005). Differential contribution of insulin receptor substrates 1 versus 2 to insulin signaling and glucose uptake in l6 myotubes.. J Biol Chem.

[32] Tanti JF, Gual P, Gremeaux T, Gonzalez T, Barres R (2004). Alteration in insulin action: role of IRS-1 serine phosphorylation in the retroregulation of insulin signalling.. Ann Endocrinol (Paris).

[33] Satoh A, Gukovskaya AS, Nieto JM, Cheng JH, Gukovsky I (2004). PKC-delta and -epsilon regulate NF-kappaB activation induced by cholecystokinin and TNF-alpha in pancreatic acinar cells.. Am J Physiol Gastrointest Liver Physiol.

[34] Schutze S, Berkovic D, Tomsing O, Unger C, Kronke M (1991). Tumor necrosis factor induces rapid production of 1′2′diacylglycerol by a phosphatidylcholine-specific phospholipase C.. J Exp Med.

[35] Liu Y, Graham C, Li A, Fisher RJ, Shaw S (2002). Phosphorylation of the protein kinase C-theta activation loop and hydrophobic motif regulates its kinase activity, but only activation loop phosphorylation is critical to in vivo nuclear-factor-kappaB induction.. Biochem J.

[36] Xu ZB, Chaudhary D, Olland S, Wolfrom S, Czerwinski R (2004). Catalytic domain crystal structure of protein kinase C-theta (PKCtheta).. J Biol Chem.

[37] Parekh D, Ziegler W, Yonezawa K, Hara K, Parker PJ (1999). Mammalian TOR controls one of two kinase pathways acting upon nPKCdelta and nPKCepsilon.. J Biol Chem.

[38] Chen LY, Doerner A, Lehmann PF, Huang S, Zhong G (2005). A novel protein kinase C (PKCepsilon) is required for fMet-Leu-Phe-induced activation of NF-kappaB in human peripheral blood monocytes.. J Biol Chem.

[39] Sun Y, Bilan PJ, Liu Z, Klip A (2010). Rab8A and Rab13 are activated by insulin and regulate GLUT4 translocation in muscle cells.. Proc Natl Acad Sci U S A.

[40] Odegaard JI, Chawla A (2008). Mechanisms of macrophage activation in obesity-induced insulin resistance.. Nat Clin Pract Endocrinol Metab.

[41] Schenk S, Saberi M, Olefsky JM (2008). Insulin sensitivity: modulation by nutrients and inflammation.. J Clin Invest.

[42] Austin RL, Rune A, Bouzakri K, Zierath JR, Krook A (2008). siRNA-mediated reduction of inhibitor of nuclear factor-kappaB kinase prevents tumor necrosis factor-alpha-induced insulin resistance in human skeletal muscle.. Diabetes.

[43] de Alvaro C, Teruel T, Hernandez R, Lorenzo M (2004). Tumor necrosis factor alpha produces insulin resistance in skeletal muscle by activation of inhibitor kappaB kinase in a p38 MAPK-dependent manner.. J Biol Chem.

[44] Li YP, Reid MB (2000). NF-kappaB mediates the protein loss induced by TNF-alpha in differentiated skeletal muscle myotubes.. Am J Physiol Regul Integr Comp Physiol.

[45] Lee JS, Yang EJ, Kim IS (2009). The roles of MCP-1 and protein kinase C delta activation in human eosinophilic leukemia EoL-1 cells.. Cytokine.

[46] Tojima Y, Fujimoto A, Delhase M, Chen Y, Hatakeyama S (2000). NAK is an IkappaB kinase-activating kinase.. Nature.

[47] Khoshnan A, Bae D, Tindell CA, Nel AE (2000). The physical association of protein kinase C theta with a lipid raft-associated inhibitor of kappa B factor kinase (IKK) complex plays a role in the activation of the NF-kappa B cascade by TCR and CD28.. J Immunol.

[48] Li YP, Atkins CM, Sweatt JD, Reid MB (1999). Mitochondria mediate tumor necrosis factor-alpha/NF-kappaB signaling in skeletal muscle myotubes.. Antioxid Redox Signal.

[49] Ji YY, Liu JT, Liu N, Wang ZD, Liu CH (2009). PPARalpha activator fenofibrate modulates angiotensin II-induced inflammatory responses in vascular smooth muscle cells via the TLR4-dependent signaling pathway.. Biochem Pharmacol.

[50] Nasser MW, Marjoram RJ, Brown SL, Richardson RM (2005). Cross-desensitization among CXCR1, CXCR2, and CCR5: role of protein kinase C-epsilon.. J Immunol.

[51] Zick Y (2003). Role of Ser/Thr kinases in the uncoupling of insulin signaling.. Int J Obes Relat Metab Disord.

[52] Aguirre V, Uchida T, Yenush L, Davis R, White MF (2000). The c-Jun NH(2)-terminal kinase promotes insulin resistance during association with insulin receptor substrate-1 and phosphorylation of Ser(307).. J Biol Chem.

[53] Nieto-Vazquez I, Fernandez-Veledo S, de Alvaro C, Rondinone CM, Valverde AM (2007). Protein-tyrosine phosphatase 1B-deficient myocytes show increased insulin sensitivity and protection against tumor necrosis factor-alpha-induced insulin resistance.. Diabetes.

[54] Hotamisligil GS, Budavari A, Murray D, Spiegelman BM (1994). Reduced tyrosine kinase activity of the insulin receptor in obesity-diabetes. Central role of tumor necrosis factor-alpha.. J Clin Invest.

[55] Wang Q, Somwar R, Bilan PJ, Liu Z, Jin J (1999). Protein kinase B/Akt participates in GLUT4 translocation by insulin in L6 myoblasts.. Mol Cell Biol.

[56] Bilan PJ, Samokhvalov V, Koshkina A, Schertzer JD, Samaan MC (2009). Direct and macrophage-mediated actions of fatty acids causing insulin resistance in muscle cells.. Arch Physiol Biochem.

[57] Hoehn KL, Hohnen-Behrens C, Cederberg A, Wu LE, Turner N (2008). IRS1-independent defects define major nodes of insulin resistance.. Cell Metab.

[58] Chin JE, Liu F, Roth RA (1994). Activation of protein kinase C alpha inhibits insulin-stimulated tyrosine phosphorylation of insulin receptor substrate-1.. Mol Endocrinol.

[59] Kawai Y, Ishizuka T, Kajita K, Miura A, Ishizawa M (2002). Inhibition of PKCbeta improves glucocorticoid-induced insulin resistance in rat adipocytes.. IUBMB Life.

[60] Mourkioti F, Rosenthal N (2008). NF-kappaB signaling in skeletal muscle: prospects for intervention in muscle diseases.. J Mol Med.

[61] Jove M, Planavila A, Laguna JC, Vazquez-Carrera M (2005). Palmitate-induced interleukin 6 production is mediated by protein kinase C and nuclear-factor kappaB activation and leads to glucose transporter 4 down-regulation in skeletal muscle cells.. Endocrinology.

[62] Kim JK, Fillmore JJ, Sunshine MJ, Albrecht B, Higashimori T (2004). PKC-theta knockout mice are protected from fat-induced insulin resistance.. J Clin Invest.

[63] Frangioudakis G, Burchfield JG, Narasimhan S, Cooney GJ, Leitges M (2009). Diverse roles for protein kinase C delta and protein kinase C epsilon in the generation of high-fat-diet-induced glucose intolerance in mice: regulation of lipogenesis by protein kinase C delta.. Diabetologia.

