# Supplementary figures and images for: Palmitate-Activated Macrophages Confer Insulin Resistance to Muscle Cells by a Mechanism Involving Protein Kinase C θ and ε
Source: PLoS One. 2011 Oct 26;6(10):e26947. doi: 10.1371/journal.pone.0026947 (PMC3202600; doi:10.1371/journal.pone.0026947)

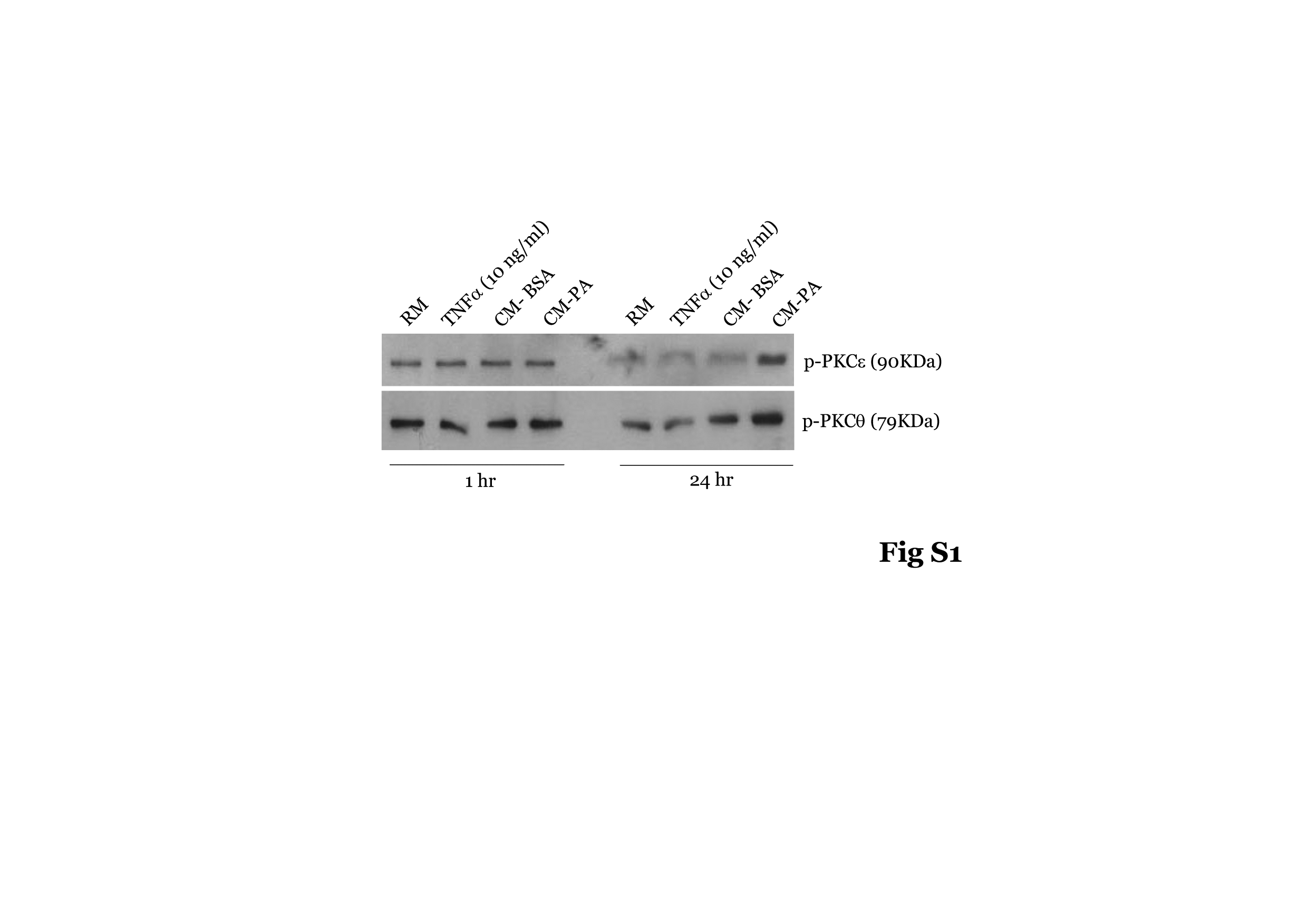

Supplement: Figure S1 — Acute incubation of muscle cells with CM-PA fails to activate novel PKC's. Macrophages were treated with BSA alone or 500 µM palmitate/1% BSA in culture medium for 6 h, then washed several times with PBS, and fresh medium was added. After 12 h, conditioned media (CM-BSA, CM-PA) were collected, centrifuged, and added to myoblast cultures for 1 h. As a control, myoblasts were simultaneously incubated with exogenous TNF-alpha (10 ng/ml for 1 h or 24 h). Lysates were prepared from all conditions and equal amounts of protein from each sample were immunoblotted with specific antibodies against phospho-PKCθ (Thr538) and phospho-PKCε (Ser729). Representative gels of two experiments are shown. (TIF) [file pone.0026947.s001.tif]

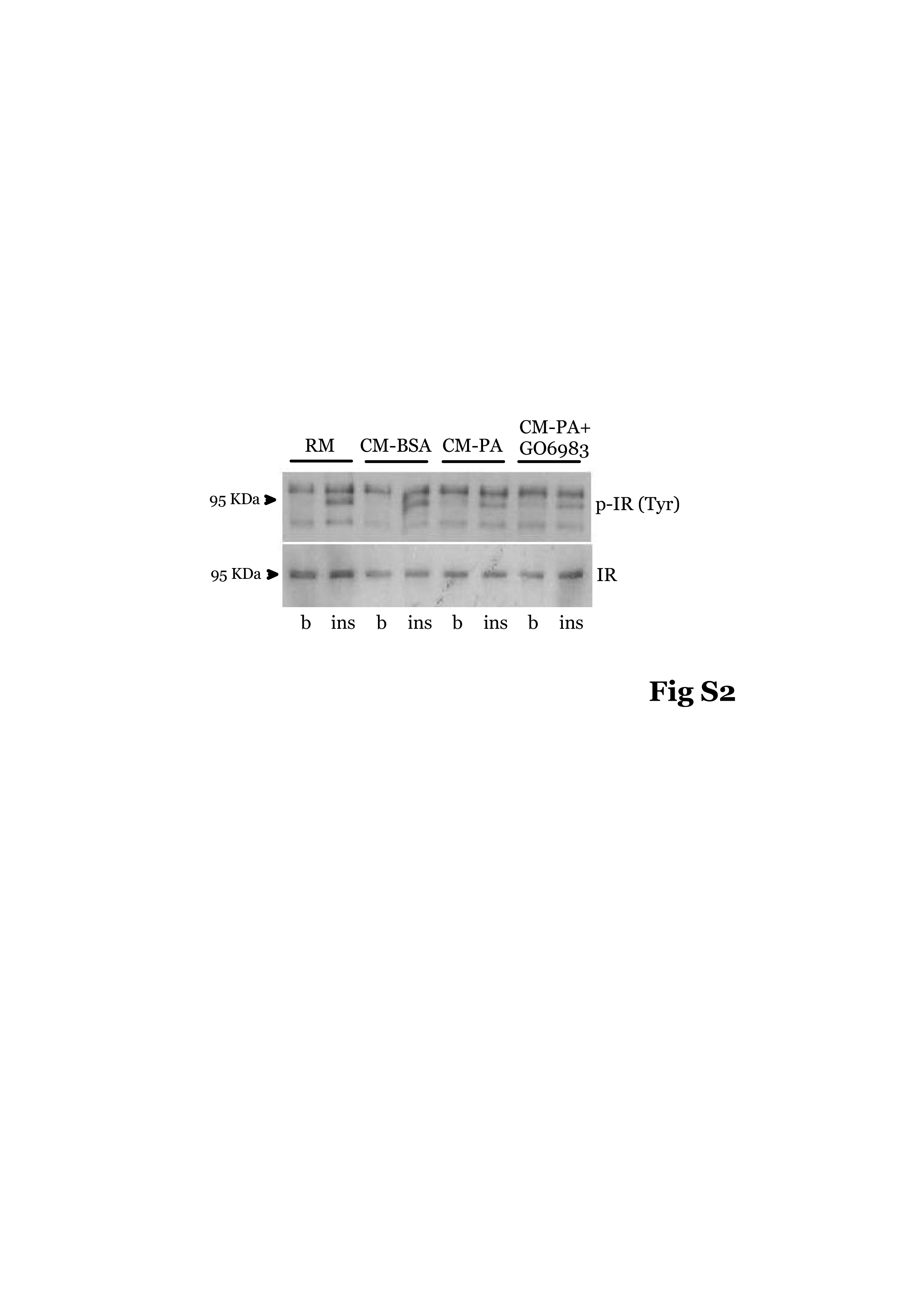

Supplement: Figure S2 — Novel PKC inhibition does not prevent the drop in insulin-stimulated tyrosine phosphorylation of the insulin receptor observed with CM-PA. L6-GLUT4myc cells expressing the human insulin receptor were treated for 24 h with RM, CM-BSA and CM-PA. Where indicated, cells were pre-incubated for 30 min with the novel and conventional PKC inhibitor, Gö6983 (1 µM), prior to addition of CM-PA. Cells were lysed and equal amount of total protein from each sample were immunoblotted with anti-pY or anti-IR β-subunit either directly or following stimulation with insulin (100 nM for 10 min). Representative gels of two experiments are shown. (TIF) [file pone.0026947.s002.tif]

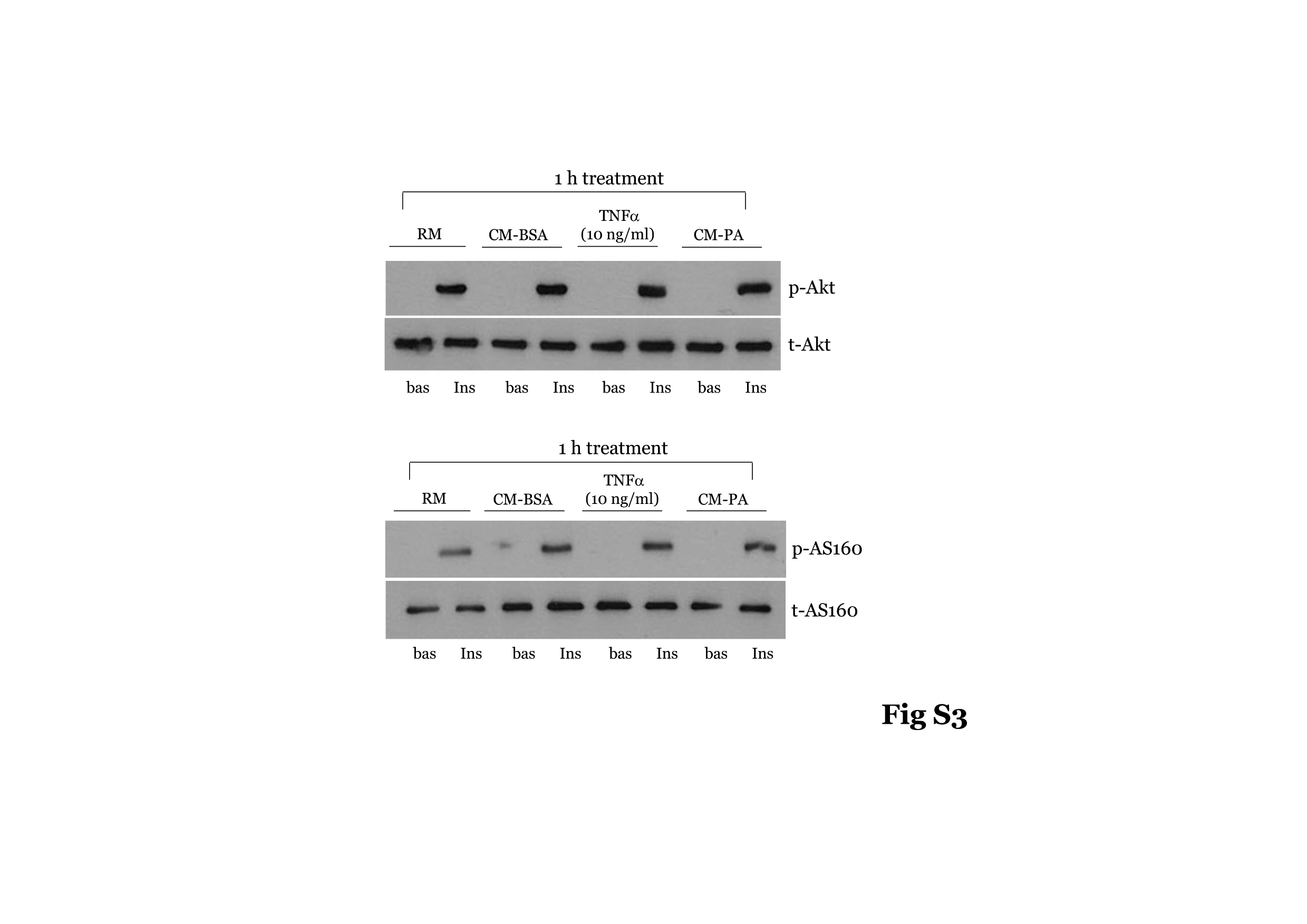

Supplement: Figure S3 — Short-term incubation of muscle cells with CM-PA does not impact on insulin-stimulated phosphorylation of Akt and AS160. Macrophages were treated with BSA alone or 500 µM palmitate/1% BSA in culture medium for 6 h, and then washed several times with PBS, and fresh medium was added. After 12 h, conditioned media (CM-BSA, CM-PA) were collected, centrifuged, and added to myoblast cultures for 1 h, then myobalsts were serum-starved and pretreated with or without insulin (100 nM, 20 min) as usual. As a control, myoblasts were simultaneously incubated with exogenous TNF-alpha (10 ng/ml for 1 h). Lysates were prepared and equal amount of protein from each sample were immunoblotted with specific antibodies against phospho-Akt (Ser473) and phospho-AS160. Representative gels of two experiments are shown. (TIF) [file pone.0026947.s003.tif]

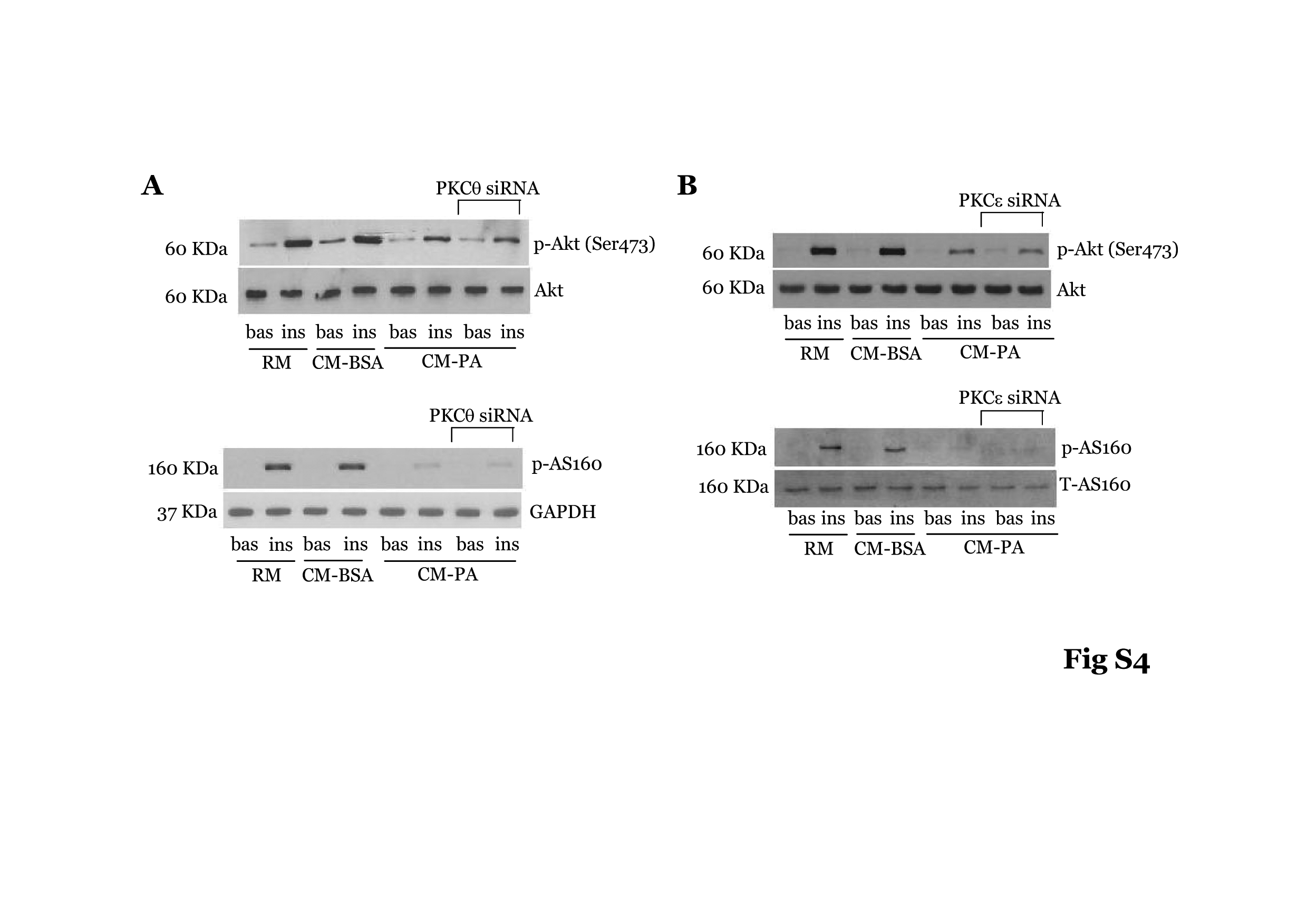

Supplement: Figure S4 — Individually silencing novel PKCθ or ε does not restore insulin sensitivity in CM-PA treated myoblasts. L6-GLUT4myc myoblasts were treated for 24 h with RM, CM-BSA, CM-PA, or CM-PA supplemented with siRNA to PKCθ or PKCε (100 nM each). Following 2 h of serum starvation, myoblasts were stimulated in the presence or absence of insulin (100 nM for 20 min). Cells were lysed, and equal amount of total protein from each sample were immunoblotted with specific antibodies against phospho-Akt (Ser473) and total Akt (A), or phospho-AS160 and total AS-160 (B). Representative immunoblots of two experiments are shown. (TIF) [file pone.0026947.s004.tif]
